# Large-scale genomic analysis of jumbo phages: coevolution, genome architecture, and host interaction mechanisms

**DOI:** 10.1186/s42523-026-00534-z

**Published:** 2026-02-24

**Authors:** Chao Wei, Zhe Chen, Yaxiang Wang, Lusheng Huang, Congying Chen

**Affiliations:** National Key Laboratory of Pig Genetic Improvement and Germplasm Innovation, Jiangxi Agricultural University, Nanchang, 330045 China

**Keywords:** Jumbo phages, Genome feature, Host bacteria, Evolution, Pigs

## Abstract

**Background:**

Jumbo phages are phages with comparatively large genome sizes. Jumbo phages have been identified in various microbial communities. However, their diversity, genome structure, potential function, and their interactions with hosts and other phages are largely unknown due to insufficient genomic data.

**Results:**

We collected 59,652,008 putative viral genomes from seven habitats by using 38 public metagenome datasets, an integrated public viral genome database (IGN), and pig gut viral genome databases. We obtained 10,754 jumbo phage genomes with sizes ranging from 200 to 831 kb. Most (94.64%) of these jumbo phage genomes were classified into Caudoviricetes, and the results have expanded the known diversity of Caudoviricetes. We found 2,389 species-like operational genome clusters that contained 3,727 (34.69%) genomes without any known viral genomes in the IGN, suggesting potential novel species-like genomes. Genome analysis suggested the potential coevolution of jumbo phages with habitat types and highlighted the utilization of alternative genetic codes and their corresponding suppressor tRNAs for recoding stop codons. CRISPR spacer analysis revealed potential bacterial or archaeal hosts of jumbo phages and uncovered competitive networks among jumbo phages. Habitat type had an important effect on the variation in phage auxiliary metabolic genes.

**Conclusions:**

This study provides an important resource and new knowledge for future studies on the interaction between jumbo phages and their bacterial or archaeal hosts.

**Supplementary Information:**

The online version contains supplementary material available at 10.1186/s42523-026-00534-z.

## Background

Phages are distinguished from eukaryotic viruses that target eukaryotic cells by specifically infecting and replicating within bacteria and archaea [1, 2]. As the most abundant biological entities on Earth, they profoundly influence global microbial ecology and evolution. However, lacking independent metabolic machinery, they must hijack the cellular processes of their specific prokaryotic hosts to reproduce [3]. Phages are ubiquitous and highly diverse, and they contribute to microecosystem balance by infecting and lysing bacterial and archaeal cells [4–6]. In addition, phages can influence the dynamics, metabolic activities, and diversity of the microbial community through several hypothetical virus-microbe interactions, such as “kill the winner,” “hitchhiking with the winner,” and “arms race” dynamics [7, 8]. Specifically, phages contribute to the maintenance of microbial diversity by selectively lysing dominant microbial strains and promoting the adaptation and diversity of host bacteria by horizontal gene transfer [9]. Additionally, phages may drive microbial diversity through adaptive co-evolution [10]. Prophages, whether potential or active, serve as the reservoirs of accessory gene pools that can enhance host survival [11]. In addition, phages directly influence host metabolism by providing auxiliary metabolic genes (AMGs) that affect key ecological processes in the environment and gastrointestinal ecosystems [12–15]. Phages also play important roles in human and animal health in applications, such as phage therapy [16, 17].

In recent years, an increasing number of studies have focused on phages, and various phage genomes have been obtained through metagenomic sequencing of the entire microbial community. However, the majority of phage genomes that have been sequenced and assembled are tens of kilobases (kb) in their whole-genome size [18–22]. Notably, some phages have a genome size > 200 kb (the largest genome size currently reported is 735 kb [3]) and are defined as jumbo phages [23]. Some studies [3, 24–26] have manually assembled complete phage genomes from metagenomic datasets to discover novel clades of ecosystem-associated jumbo phages. However, the limited number of both cultured and sequenced jumbo phage genomes in public repositories has hampered comprehensive assessment of the ubiquity, diversity, and distribution of jumbo phages in nature. Current researches have indicated that jumbo phages, notably due to their large genome size, encode a rich repertoire of auxiliary metabolic genes (AMGs). These AMGs have also been identified in various phages and are involved in the metabolism processes, such as methane oxidation (pmoC) and NAD+ synthesis. They have been though to modulate host metabolism and improve the fitness of host microbes during infection [25, 27]. Previous reports also suggest that jumbo phages may use alternative codon patterns to increase their fitness [22, 28–30]. Specifically, translation in phages could bypass the conventional stop codons (TGA, TAA, and TAG) to maintain the integrity of proteins by using alternative genetic codes (codes 15, 90, and 91). Previous studies have shown that some jumbo phages have intact and functional CRISPR-Cas systems that could target host defenses as well as other phages competing for the same host [3, 31–34]. In particular, several phages with genome size > 500 kb (megaphages) have been predicted to infect *Prevotella* species based on CRISPR spacer matches and were enriched in human and animal gut microbiomes [24, 35, 36]. However, the number of jumbo phages reported in animal guts remains few, mainly due to technical challenges in the assembly of their genomes with metagenomic sequencing data, including short sequence reads of metagenomes with second generation sequencing, repetitive or complex genomic regions, and low abundance of jumbo phages in samples [27, 37]. The general characteristics of jumbo phages in the animal guts or other habitats has not largely uncovered.

To characterize the composition and diversity of jumbo phages, we first identified jumbo phage genomes from a pig gut viral genome database constructed in our lab and seven metagenomic sequencing datasets of the pig gut microbiome downloaded from public repositories. In addition, to compensate for the insufficient number of jumbo phage genomes and to assess the prevalence and distribution of jumbo phages in different microbial ecosystems, we collected 38 public viral genomes from seven habitats and integrated the viral genomes from four existing public viral genome databases: IMG/VR [38], GenBank [39], and Nucleotide Sequence Database (NT) [40]. These datasets were used to construct a jumbo phage genome dataset containing 10,754 genomes. Based on this, we characterized the composition, lifestyle, genomic structure, and distribution of jumbo phages from different habitats. Specifically, we investigated alternative genetic codes, host bacteria, phage-phage competition networks, potential functions, and novel clades in jumbo phages. The results provide a powerful resource and a reference for extensive studies of jumbo phages.

## Methods

### Collection of viral genome datasets and the construction of a jumbo phage genome database

We first collected viral genomes from the PGV genome database using shotgun metagenomic sequencing reads and Pacbio HiFi long-read sequencing data from our lab. To expand the known diversity of viral genomes from the pig gut, we downloaded raw metagenomic sequencing data from seven previously published studies of pig gut microbiomes from the NCBI Sequence Read Archive database (https://www.ncbi.nlm.nih.gov/sra), and 442,287 contigs were assembled using Megahit (v1.2.9) [41] with the default parameters. Additionally, we collected viral genomes from 38 published studies and an integrated viral genome database (IGN). These 38 published studies contained viral genomes from seven habitat types: (1) the rumen, (2) the pig gut, (3) non-human primate guts, (4) the human gut, (5) soil and lake sediments, (6) freshwater and meltwater, and (7) oceans. The integrated viral genome database included dereplicated viral genomes collected from the IMG/VR, GenBank, and NT databases. Finally, 59,652,008 putative viral genomes were retained for further analysis.

We used a multi-step bioinformatic pipeline to identify high-confidence phage contigs from these putative viral genomes. First, putative viral contigs were identified from the initial 59,652,008 contigs using the criteria established by Nayfach et al. [28], which combined four signatures: the presence of viral protein families, the absence of conserved microbial protein families, nucleotide composition signatures of viral genomes, and the organization of multiple adjacent viral genes on the same strand. Subsequently, putative viral contigs with the length < 3 kbp were removed from further analysis. To distinguish phage genomes from other viral genomes within the obtained viral genome dataset, we employed two complementary methods: (1) Signature gene-based filtering: viral contigs were required to include at least two genes with virus-specific keywords (e.g., “capsid, phage, terminase, base plate, baseplate, prohead, virion, virus, viral, tape measure, tapemeasure neck, tail, head, bacteriophage, prophage, portal, DNA packaging, T4, p22, and holin”). To reduce the contamination from prokaryotic genomic fragments, contigs containing genes annotated with prokaryote-specific keywords including “ribosomal protein, preprotein translocase, and DNA gyrase subunit A” were excluded. In addition, potential phages must be matched by at least one spacer predicted from bacterial and archaeal genomes. (2) Machine learning-based prediction: The refined phage genome set was reanalyzed using a deep learning model of PhaMer (v1.0) [42] with the default parameters, which was trained to identify phage sequences. Finally, the identified phage genomes were categorized using the conventional phage genome database (CPGD, genome sizes ≤ 200 kb) and jumbo phage genome database (JPGD, genome sizes > 200 kb). In addition, we used CheckV (v0.8.1) [43] to detect proviruses boundaries, remove contamination by host bacterial sequences, and evaluate the completeness of the phage genomes.

### Removing false positives of jumbo phage genomes

Although it has always been difficult to distinguish some viral and bacterial contigs, as bacterial genomes may harbor genes from phages infecting them and viral genomes may carry bacterial genes obtained from their hosts, we assessed the distribution of bacterial and viral genes in each putative jumbo phage genome to remove possible false positives (contigs). The distribution of bacterial genes in putative jumbo phage genomes was assessed by counting the number of hits to highly conserved genes of bacterial universal single-copy orthologs (BUSCOs). Briefly, protein sequences encoded by phage genes were compared against 318 BUSCO gene HMMs using hmmsearch with the “-E 0.05” option, and then, the BUSCO-provided HMM score cut-offs were used to filter the hits. BUSCO ratios ranging from 0 to 0.067 derived from viral genomes in the Viral RefSeq and established by Gregory et al. [44] were considered acceptable baselines. We then assessed the BUSCO ratios for all jumbo phage genomes and compared the results to the Viral RefSeq BUSCO ratios. Only the jumbo phage genomes with a BUSCO ratio of < 0.067 were retained in the database. In addition, we performed a competitive search for bacterial-specific protein HMMs, archaeal-specific protein HMMs, mixed protein HMMs, and viral-specific protein HMMs with Virsorter2 using hmmsearch with the “-T 30” option and manually assessed the distribution of viral and bacterial genes in putative jumbo phage genomes.

### Lifestyle prediction and taxonomy assignment of jumbo phages

The BACPHLIP (v0.9.3) program [45] was used to distinguish virulent and temperate phages based on the identified jumbo phage genomes using a scoring system. Jumbo phage genomes were classified into three categories: temperate (those with scores > 0.9), uncertain (those with scores in the range of 0.5–0.9), and virulent (those with scores < 0.5). Because temperate phages can exist in both lytic and lysogenic status, prophages identified using CheckV (v0.8.1) and temperate phage genomes detected using BACPHLIP were classified as temperate phages. For the taxonomic annotation of jumbo phage genomes, we used geNomad (v1.7.4), which assigns taxonomic labels to jumbo phage genomes based on a database of marker genes from viral groups encompassing lineages related to those recognized by the ICTV [46].

### Clustering jumbo phage genomes at the species-like, genus-like, and family-like operational clusters and identification of potential novel jumbo phage genome clusters

All 10,754 reconstructed jumbo phage genomes were clustered into species-like operational clusters using a stringent sequence similarity threshold of ≥ 95% average nucleotide identity (ANI) and ≥ 85% of the genome coverage using the algorithm described by Nayfach et al. [28]. And then, jumbo phage genomes were further organized into genus-like and family-like operational clusters based on average amino acid identity (AAI) and shared genes. Briefly, proteins predicted from all phage genomes using Prodigal were aligned with BLASTP in DIAMOND (v2.1.9.163). For each genome pair, we calculated the AAI and the percentage of shared genes. Genome pairs with > 50% AAI or > 20% shared genes were grouped into genus-like operational clusters using the MCL algorithm (v14-137) with an inflation factor of 2.0, and genome pairs with > 20% AAI or > 10% shared genes were clustered into family-like operational clusters using MCL with an inflation factor of 1.2. These operational clusters were used for structuring the phage genome dataset. Those clusters that did not contain jumbo phage genomes from the IGN were considered novel jumbo phage genome clusters. The potential novel jumbo phage genome clusters were analyzed at the species-like, genus-like, and family-like clustering levels.

### Potential coevolution analysis of jumbo phages with habitat types

A phylogenetic tree was constructed for each genus-like jumbo phage genome cluster that contained at least four genomes and existed in at least two habitat types [18]. Briefly, protein-coding genes were initially predicted from the jumbo phage genomes. Core genes of each genus-like jumbo phage genome cluster were identified using Roary (v1.7.8) [47] with the “-i 50” option, and a multi-FASTA alignment was created. Phylogenetic trees were built using FastTree (v2.1.10) [48] with the default parameters and visualized and annotated using iToL (https://itol.embl.de/). The branch lengths between any two genomes were then calculated for each cluster. The branch lengths between jumbo phage genomes from the same habitat types and those from different habitat types were compared using two one-tailed Wilcoxon rank sum tests. If the branch lengths in a genus-like genome cluster from different habitat types were significantly (*P* < 0.05) longer than those from the same habitat types, we considered that this genus-like jumbo phage genome cluster had coevolved with the habitat type.

### Identifying alternative genetic codes in jumbo phage genomes

Prodigal (v2.50) was used to identify open reading frames (ORFs) of protein-coding genes in 10,754 jumbo phage genomes (JPGD) under the standard genetic code (code 11) and three alternative genetic codes: TAG recoding (code 15), TAA recoding (code 90), and TGA recoding (code 91) [28, 49, 50]. Briefly, for a jumbo phage genome, if the protein-coding density under genetic code 15, 90, or 91 was increased by 10% compared with that under the standard genetic code (11), we considered that this jumbo phage genome used the corresponding alternative genetic code.

### Host prediction for jumbo phages and identification of phage-phage interactions

The hosts of jumbo phages were predicted based on CRISPR spacer matching. CRISPR spacers were identified from microbial genomes (or MAGs) in the GTDB, UHGG, and PGV datasets using MinCED (https://github.com/ctSkennerton/minced) with default options, and the taxonomic information of MAGs was annotated using GTDB-tk [51] with the “classify_wf mode” option. All CRISPR spacers were then mapped to jumbo phage genomes using blastn with the “-max_target_seqs 10000000 -dust no -word_size 8 -evalue 10” option. If a CRISPR spacer was mapped to a jumbo phage genome with a maximum of one mismatch and 100% sequence alignment, that microbial genome or MAG indicated that this CRISPR spacer was from the host of the corresponding jumbo phage. To identify pathogenic bacteria from jumbo phage hosts, amino acid sequences were extracted from host bacterial genomes and aligned against the PHI database (http://www.phi-base.org/) using Diamond BLASTP with the parameters “-e 1e-5 --id 80 -k 10 -b 8.”

Similarly, all jumbo phage genomes were searched against the CRISPR spacers predicted from the jumbo phage genomes to identify phage-phage interactions. Briefly, all jumbo phage genomes were processed using MinCED with the default options to identify CRISPR spacer arrays and then queried with blastn against CRISPR spacers. Hits were retained if a viral CRISPR spacer was mapped to another jumbo phage genome with a maximum of one mismatch and 100% sequence alignment. CRISPR-Cas systems in jumbo phage genomes and their hosts were predicted using the CRISPRCasTyper with the “-mNS 2” option. Cas and CRISRP-associated proteins were also predicted based on the custom HMM profiles using hmmsearch with the setting “-E 1e-5.” Anti-CRISPR proteins (Acrs) in jumbo phage genomes and their hosts were identified using Acafinder with the “-l 800 -i 300 -b 10” option. Defense systems of phage hosts were predicted using DefenseFinder with the default parameters.

### Functional annotation of jumbo phage genomes

Proteins encoded by jumbo phage genomes in the JPGD were annotated based on the Pfam-A [52], TIGRFAM [53], and VOGDB (http://vogdb.org) databases using hmmsearch [54] with the “-E 1e-5” option. Considering that some proteins might not be annotated by hmmsearch, to improve the functional annotation, we also blasted protein sequences to the jumbo phage database *TerL* and major capsid proteins using blastp with the “--min-score 50 -e 1e-5” option.

Antibiotic resistance genes (ARGs) in jumbo phage genomes were identified using three tools: (1) the Resistance Gene Identifier (RGI, v.5.1.0) with gene-specific bit-score thresholds, (2) the AMRFinder tool (amrfinderplus, v.3.8.4) with the default parameters, and (3) the ResFinder tool (v4.0) with the “-l 0.6 --acquired” option. In addition, potential phage-encoded virulence factors (VFGs) were identified using BLASTp alignment against the VFDB (VFDB_setB_pro.fas) file. A protein was annotated as a potential VF when its best hit had ≥ 60% identity and ≥ 70% query coverage.

AMGs in jumbo phage genomes were detected using VIBRANT (v1.2.1) [55] and DRAM-v (v1.3.5) [56]. Briefly, proteins encoded by phage genomes were first scored by VirSorter2 (v2.2.2) [57], and the scored proteins were annotated using DRAM-v with the default options. AMGs were also annotated using VIBRANT and assigned to metabolic pathways using the KEGG database [58]. Only those AMGs annotated by both VIBRANT and DRAM-v were retained for further analysis. We used ColabFold, which combines a fast homology search by MMseqs2 (v2.0) [59] with AlphaFold2 (v2.0) [60] to predict the three-dimensional structures of AMGs. Five AlphaFold2 models were generated for each protein, and the highest-ranked model was used for structural alignment. Visualization, superposition, and RMSD value calculation were performed using ChimeraX (v1.7) [61] with the default parameters.

### Construction of phylogenetic trees for jumbo phage genomes of Caudoviricetes

A phylogenomic tree for the Caudoviricetes genomes was constructed using the method described by Low et al. [62]. First, we identified a subset of 77 gene markers from the predicted protein sequences of Caudoviricetes genomes based on individual searches against the HMM profiles of these 77 gene markers using HMMER. We then trimmed and concatenated all identified marker gene alignments to retain those genome fragments with less than 50% gaps using trimAl (v1.4.rev22) [63]. Only those viral genomes that contained at least three marker genes and were present in > 5% of alignment columns were retained. Finally, a phylogenetic tree was constructed using IQ-TREE2 (v2.1.3) [64] with 1,000 bootstrap iterations and visualized using iTOL (https://itol.embl.de/) [65].

### Statistical analysis

All statistical analyses were performed using R packages (v4.2.1).

## Results

### Construction of jumbo phage genome database (JPGD) using viral genomes from different habitats

**Fig. 1:**
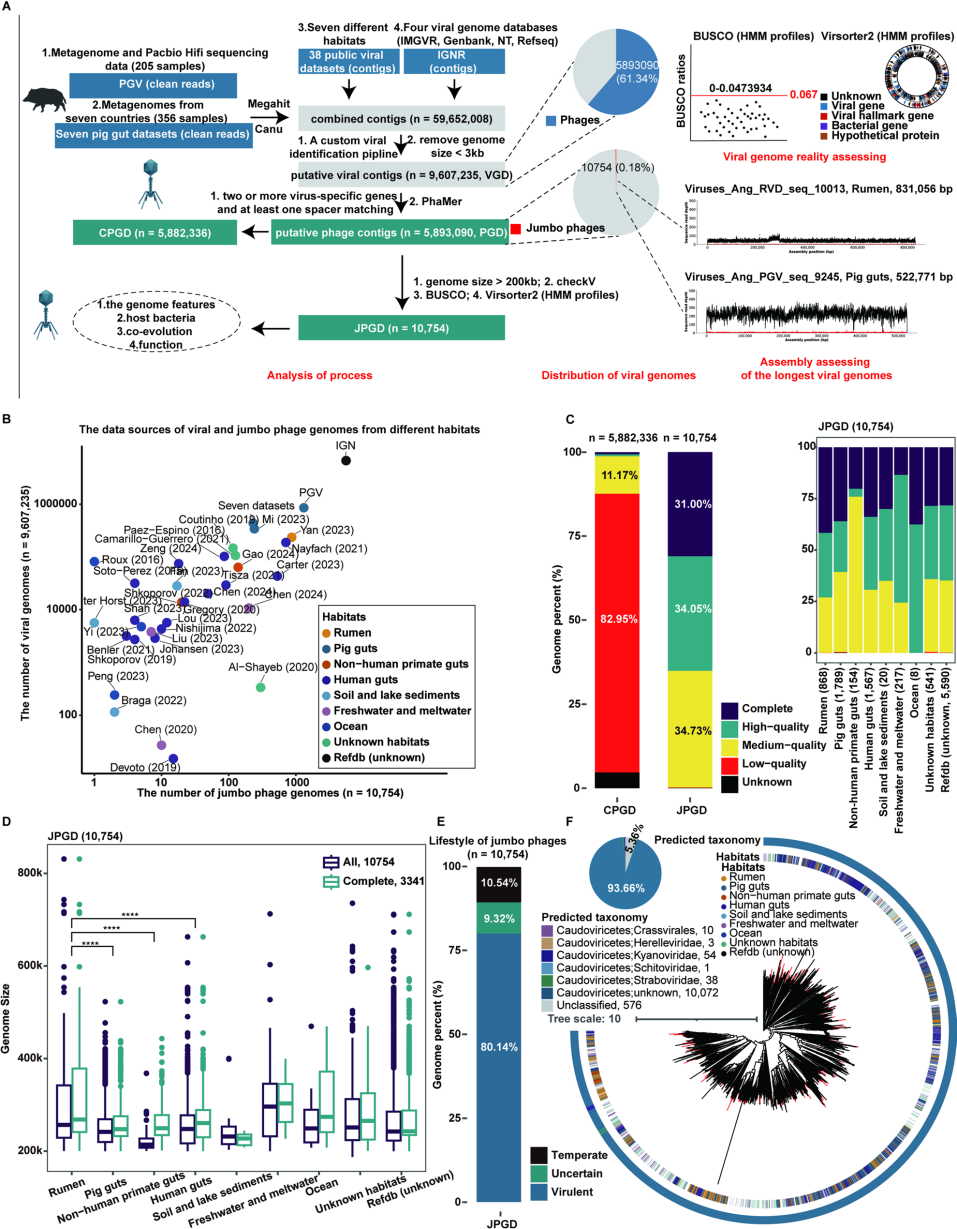
Identification and characterization of jumbo phage genomes from different habitats. (**A**) An overview of the identification and assessment of phages and jumbo phages. (**B**) The data sources for viral and jumbo phage genomes from different habitats. The *x*-axis shows the number of jumbo phage genomes, and the *y*-axis shows the number of viral genomes. (**C**) The quality distribution of phage genomes in the JPGD and CPGD (left) and among different habitats (right). The stacked bars with different colors represent the completeness of phage genomes. (**D**) A comparison of phage genome sizes in the JPGD among different habitats. (**E**) The lifestyles of 10,754 jumbo phage genomes in the JPGD. The different colors represent different lifestyles in the stacked bars. **(F**) The taxonomy of 10,754 jumbo phage genomes from different habitats in the JPGD. The pie chart shows the taxonomy of jumbo phages, and different colors represent different viral taxonomies. The phylogenetic tree constructed based on 77 marker genes shows the distribution of jumbo phages in the Caudoviricetes class, and the outer circles represent the taxonomy and habitat type of jumbo phages

To characterize the composition and diversity of jumbo phages, we identified genomes or genome fragments of jumbo phages from the PGV genome database constructed in our lab. We also recovered genome fragments of jumbo phages from metagenomic sequencing data of pig fecal samples from seven countries (Additional file 2: Table S1 and Additional file 1: Figure S1A). Furthermore, to increase the coverage of the JPGD, viral genomes from 38 public datasets spanning seven different habitats and from an integrated viral database (IGN) including the IMG/VR, GenBank, and NT databases were collected. We obtained a total of 9,607,235 putative viral genomes through a viral genome identification pipeline based on the assembled viral genomes and contigs using Megahit. An integrated approach combining machine learning, spacer sequence matching, and virus-specific gene identification was used to identify phage genomes from the viral genomes. A total of 5,893,090 phage genomes (hereafter the phage genome database, PGD) were obtained and used for further analysis. Based on their genome sizes, these phage genomes were further divided into a JPGD containing 10,754 phages with genome sizes > 200 kb and a CPGD comprising 5,882,336 phages with genome sizes ≤ 200 kb (Fig. 1A). Among the 10,754 jumbo phage genomes, 110 with genome sizes > 500 kb were classified as megaphages.

Jumbo phage genomes were identified in each of the viral genome datasets from seven habitats (Additional file 3: Table S2). The number of viral and jumbo phage genomes varied significantly, with more jumbo phage genomes identified from the pig gut, the human gut, and the rumen (Fig. 1B), highlighting the widespread prevalence of jumbo phages in diverse environments. Interestingly, more jumbo phage genomes were identified in the PGV viral genome dataset than in the PGV genome datasets from repository databases (*n* = 1,314, Additional file 1: Figure S1B). This could be attributed to the utilization of an ultra-sequencing depth, Pacbio long-read sequencing, or extensive sample sources across various pig cohorts, gut locations, and age stages.

We then evaluated the completeness of all phage genomes in the PGD (*n* = 5,893,090) using CheckV (v0.8.1) and found that the JPGD predominantly contained phage genomes of medium to high quality (*n* = 10,731, 99.78%). In contrast, the CPGD was dominated by phage genomes with low to medium quality (*n* = 5,536,928, 94.12%). However, there were no significant differences in the completeness of jumbo phage genomes among the seven habitat types, with 50.27–100% completeness for most genomes (Fig. 1C). The largest genome sizes for jumbo phages from the rumen, human gut, non-human primate gut, and pig gut microbiome were 831,056 bp, 662,832 bp, 368,231 bp, and 522,771 bp, respectively (Fig. 1D). To exclude possible false assembly caused by multiple repetitions of the genomes, we mapped the clean reads to the jumbo phage genomes with the largest genome size in the JPGD to assess the uniformity of sequence coverage using the metagenomic sequencing dataset in which the jumbo phage genome was detected. The results showed that these jumbo phage genomes exhibited perfect coverage uniformity, indicating that the assemblies were reliable and without errors (Additional file 1: Figure S1B).

CheckV (v0.8.1) and BACPHLIP (v0.9.3) were used to predict the lifestyles of 10,754 jumbo phages. Jumbo phages in the JPGD had a higher proportion of virulent jumbo phages than temperate phages (*n* = 8,618, 80.14%, Fig. 1E). Interestingly, prophage genomes (*n* = 252, 2.34%) belonging to jumbo phages were identified across habitat types, suggesting extensive interaction between jumbo phages and bacteria. To further evaluate the taxonomic composition of jumbo phage genomes in the JPGD, we annotated 10,754 genomes using geNomad (v1.7.4) classification software. Only 106 jumbo phage genomes were classified into known families (Additional file 4: Table S3), with the rest (*n* = 10,072, 93.66%) only being assignable to Caudoviricetes, a class of double-stranded DNA (dsDNA) phages (Fig. 1F). These findings not only underscore the novelty of jumbo phages in the JPGD but also highlight the challenges in their classification.

### Expanded diversity of jumbo phages and potential coevolution with habitat types

**Fig. 2:**
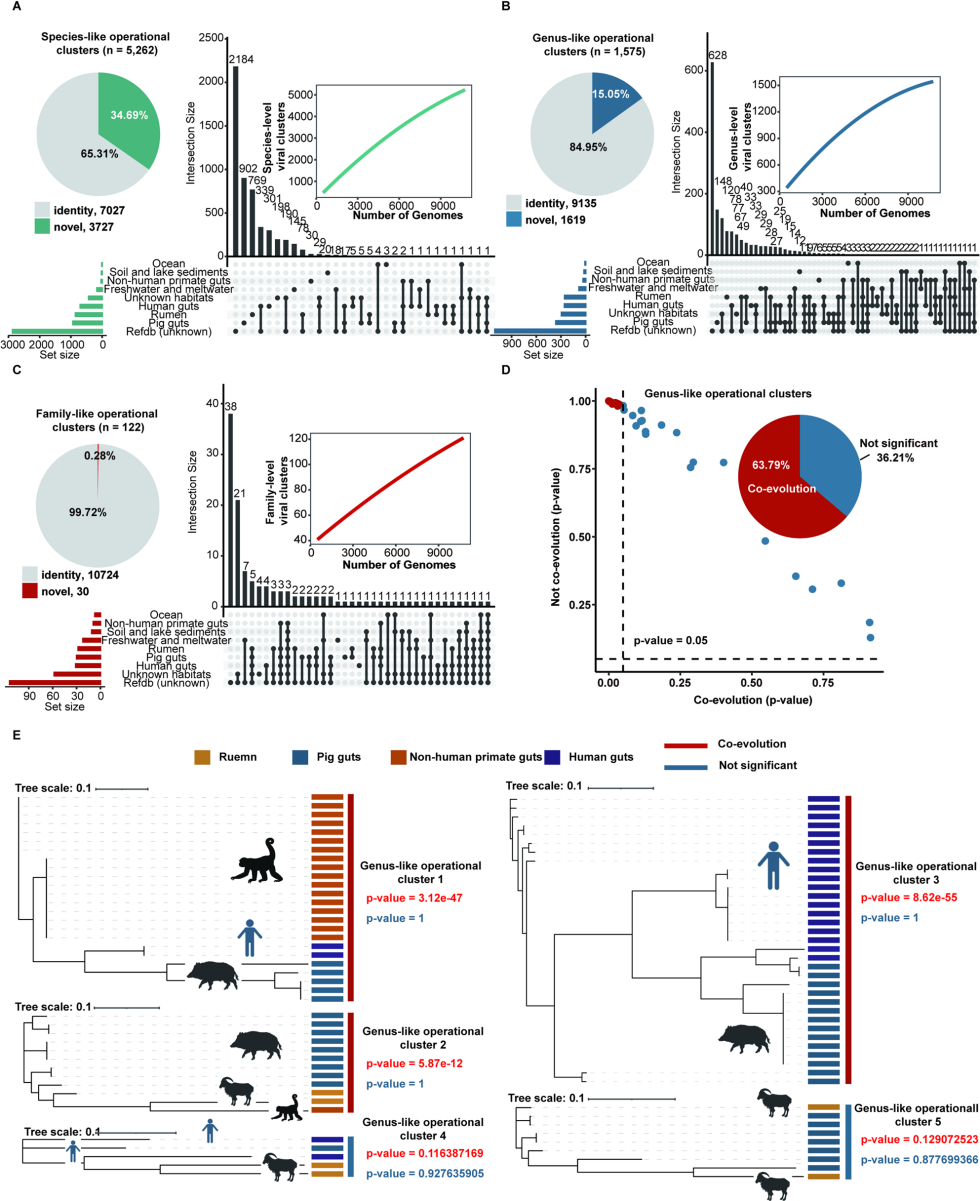
Expanded diversity and potential coevolution of jumbo phages with habitat types. (**A–C**) The number of novel jumbo phages identified in this study and the distribution of jumbo phages among different habitats at various taxonomic levels. The pie charts show the proportions of novel and known phage genomes in the JPGD. The bar graphs represent the number of jumbo phages unique or shared among different habitat types. Accumulation curves indicate the relationship between the number of jumbo phage genomes and the number of species-like operational clusters, genus-like operational clusters, and family-like operational clusters identified. (**D**) Potential coevolution analysis of genus-like operational clusters of jumbo phages in different habitat types. The dot plot shows the likelihood (*P* values) distributions of 58 genus-like operational clusters that were potentially coevolved (red dots) or not coevolved (blue dots) with habitat types. The pie chart shows the proportions of potentially coevolved and not coevolved genus-like operational clusters. (**E**) Three examples of genus-like operational clusters of jumbo phages that potentially coevolved with habitat types and two examples of genus-like operational clusters of jumbo phages that did not coevolve with habitat types

To evaluate the diversity of jumbo phages in the JPGD, 10,754 jumbo phage genomes were clustered into 5,262 species-like operational clusters using the threshold criteria of 95% average nucleotide identity (ANI) and 85% coverage. The results showed that 902 (17.14%), 769 (14.61%), 30 (0.57%), and 301 (5.72%) species-like operational clusters were specifically identified in the pig gut, rumen, non-human primate gut, and human gut, respectively. To identify those jumbo phage genomes first reported in this study, all jumbo phage genomes were clustered in the JPGD at 95% ANI and 85% coverage. We found that 3,727 (34.69%) jumbo phage genomes belonging to 2,389 (45.40%) species-like operational clusters in the JPGD were not clustered with any jumbo phage genomes in the IGN, suggesting that these were novel jumbo phage genomes identified in this study (Fig. 2A). We further clustered jumbo phage genomes in the JPGD into genus-like operational clusters at the threshold of > 50% average AAI or > 20% of shared genes and an inflation factor of 2.0, and into family-like operational clusters at > 20% AAI or > 10% shared genes under an inflation factor of 1.2. A total of 1,575 genus-like operational clusters and 122 family-like operational clusters were obtained. Among these, 148 genus-like operational clusters (9.40%) and one family-like operational cluster (0.82%) of jumbo phages from the pig gut and 33 genus-like operational clusters (2.10%) and one family-like operational cluster (0.82%) from the human gut did not contain any jumbo phage genomes from the IGN or the datasets for other habitat types, suggesting novel candidates of jumbo phage genera and a family specifically identified in the pig gut and human gut (Fig. 2B and C). Furthermore, accumulation curves for the numbers of family-like operational clusters, genus-like operational clusters, and species-like operational viral clusters obtained did not achieve saturation for jumbo phages using the JPGD (Fig. 2A–C), indicating the high diversity of jumbo phages and that more samples from wider sources would be needed to cover additional taxa.

We then evaluated whether the jumbo phage genomes had potentially coevolved with habitat types using genus-like operational clusters. Considering the high diversity of jumbo phages, 58 genus-like operational clusters containing at least four jumbo phage genomes and existing in at least two habitat types were included in the analysis. For each genus-like operational cluster, we evaluated whether jumbo phage genomes from the same habitat type were more closely related than genomes from different habitat types by constructing a phylogenetic tree as described by Wu et al. [18]. The jumbo phage genomes in a genus-like operational cluster from the same habitat type were clustered in the phylogenetic tree and had significantly shorter evolutionary distances than jumbo phage genomes from different habitat types in more than 63.79% of the genus-like operational clusters (*n* = 37, Additional file 5: Table S4), suggesting potentially coevolution of jumbo phages with their habitat types (Fig. 2D). As an example, the jumbo phage genomes in the same genus-like operational cluster (genus-like operational clusters 1, 2, and 3) from the same habitat type had significantly shorter phylogenetic distances than those from different habitats. However, this potentially coevolutionary relationship was not observed for the jumbo phage genomes from genus-like operational clusters 4 and 5, suggesting heterogeneity of potential coevolution among jumbo phage taxa (Fig. 2E).

### Highlighting the reassignment of stop codons and the corresponding suppressor tRNAs in jumbo phage genomes

**Fig. 3:**
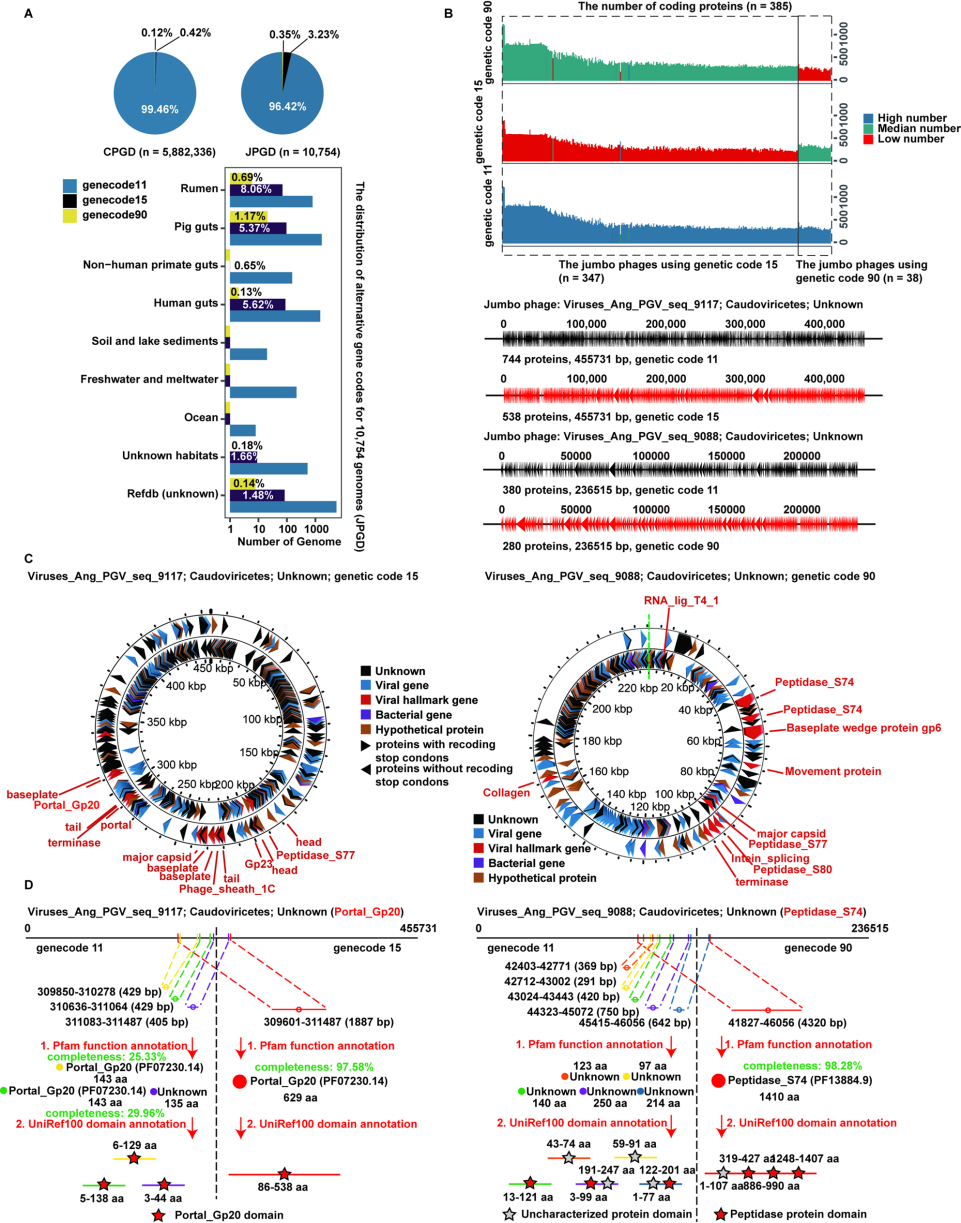
Utilization of alternative genetic codes in jumbo phage genomes. (**A**) The proportion and distribution of alternative genetic codes in phage genomes from the JPGD and CPGD. Different colors represent different genetic codes, and the pie charts show the proportions of phages using alternative genetic codes in the JPGD and CPGD. The bar plots show the distribution of alternative genetic codes in 10,754 jumbo phage genomes from different habitats. (**B**) The numbers of coding proteins for 385 jumbo phages using alternative genetic codes. The *x*-axis shows the jumbo phage genomes using alternative genetic codes, and the *y*-axis shows the number of coding proteins for each genome under different genetic codes. The line plots show the comparison of coding proteins of jumbo phage genomes using the standard genetic code and alternative genetic codes. (**C**) Circle diagrams highlighting the genes predicted by the recoding of stop codons. The outer circle represents genes using the recoding of stop codons, and the inner circle shows genes using standard stop codons. Different colors represent different gene types. (**D**) A comparison between coding proteins using an alternative genetic code and the standard genetic code. The structure, Pfam-A function annotation, and domain annotation with UniRef100 are compared

Low protein-coding density [24] and the fragmentation of many predicted proteins were distinctive features of jumbo phage genomes, suggesting the utilization of alternative genetic codes by jumbo phages. To assess the recoding of stop codons in jumbo phage genomes (JPGD), a Prodigal (v2.50) program was used [24] to select appropriate genetic codes (genetic codes 11, 15, 90, and 91) based on the total potential coding scores of the genetic codes. Although the standard genetic code (genetic code 11) was used in approximately 99.46% and 96.42% of the phages in the JPGD and CPGD, respectively, the stop codons TAG and TGA were treated as the codons for glutamine (Q) and Glycine (G), respectively (defined as genetic codes 15 and 90), in a small proportion of phages (Fig. 3A). Notably, no jumbo phages recoded the stop codons of TAA as glutamine (Q) (defined as genetic code 91) in the JPGD or CPGD. A higher proportion (3.58%, *n* = 385) of jumbo phages in the JPGD appeared to use alternative genetic codes than phages (0.54%) in the CPGD. Additionally, higher proportions of jumbo phages from the rumen (8.75%), pig gut (6.54%), and human gut (5.75%) used alternative genetic codes than jumbo phages from other habitat types (0–0.65%).

The protein-coding density analysis of jumbo phage genomes showed that the numbers of predicted coding proteins were significantly higher when genetic code 11 was used in the ORF analysis rather than genetic code 15 or 90, except for two jumbo phage genomes that had a higher number of coding proteins when genetic code 15 or 90 was used (Fig. 3B). Furthermore, there were 38 jumbo phage genomes in which more coding proteins were identified when genetic code 15 was used rather than genetic code 90. The result was reversed for the other 383 jumbo phage genomes. We predicted the lifestyles of 385 jumbo phages that used the alternative genetic codes; 4.94% of these jumbo phages were temperate phages; 82.08% were virulent phages; and the other 12.98% were uncertain (Additional file 1: Figure S2A).

We further investigated whether stop codon recoding was associated with specific types of proteins. The results indicated that recoding of the stop codons TAG and TGA did not exist in most regions of the jumbo phage genome but occurred in many viral hallmark gene regions (Fig. 3C), and there was no preference for specific protein types. To confirm the reliability of stop codon recoding, we checked the proteins translated by these hallmark genes. As an example of the utilization of genetic code 15, the *Portal_GP20* gene encoded three small peptides when the standard genetic code (11) was used; these were fragmented, and only two peptides with very low protein completeness (25.33% and 29.96%) were annotated by Pfam-A. Furthermore, the structural domains of these three small peptides were incomplete, with the UniRef100 program indicating fragmentation. However, when genetic code 15 was used, this gene encoded a *Portal_GP20* protein with 97.58% completeness and a complete structural domain (Fig. 3D). As another example, using the standard genetic code (11), the *Peptidase_S74* gene encoded five small peptides that were highly fragmented and could not be accurately annotated by Pfam-A. The structural domains of these five small peptides were also fragmented. However, using genetic code 90, the encoded *Peptidase_S74* protein contained 1,410 amino acids (protein completeness: 98.28%) that could be functionally annotated using Pfam-A, and its typical peptidase protein domain could also be annotated using UniRef100 (Fig. 3D), confirming the reliability of the stop codon recoding.

Phages using repurposed stop codons usually encode a suppressor tRNA [24]. We screened tRNAs and suppressor tRNAs encoded by jumbo phage genomes utilizing alternative genetic codes using tRNAscan-SE (v2.0.9). More than 88.31% and 60.78% of jumbo phage genomes encoded tRNAs and suppressor tRNAs, respectively (Additional file 1: Figure S2B). The jumbo phages with recoding of the stop codon TAG (code 15) could encode a suppressor tRNA with a CTA anticodon necessary for recoding the stop codon TAG. Meanwhile, some jumbo phage genomes also contained genes encoding the release factor 2 that could terminate translation by recognizing the stop codons TGA and TAA but not TAG. As described in a previous study [24], we also recognized intronic sequences in tRNAs encoded by jumbo phage genomes (*n* = 262, 77.06%) using alternative genetic codes (Additional file 1: Figure S2C). These internal introns of tRNAs could encode another tRNA. The results highlighted the alternative genetic codes used in jumbo phages and their corresponding suppressor tRNAs.

### CRISPR spacer analysis reveals the potential hosts of jumbo phages

**Fig. 4:**
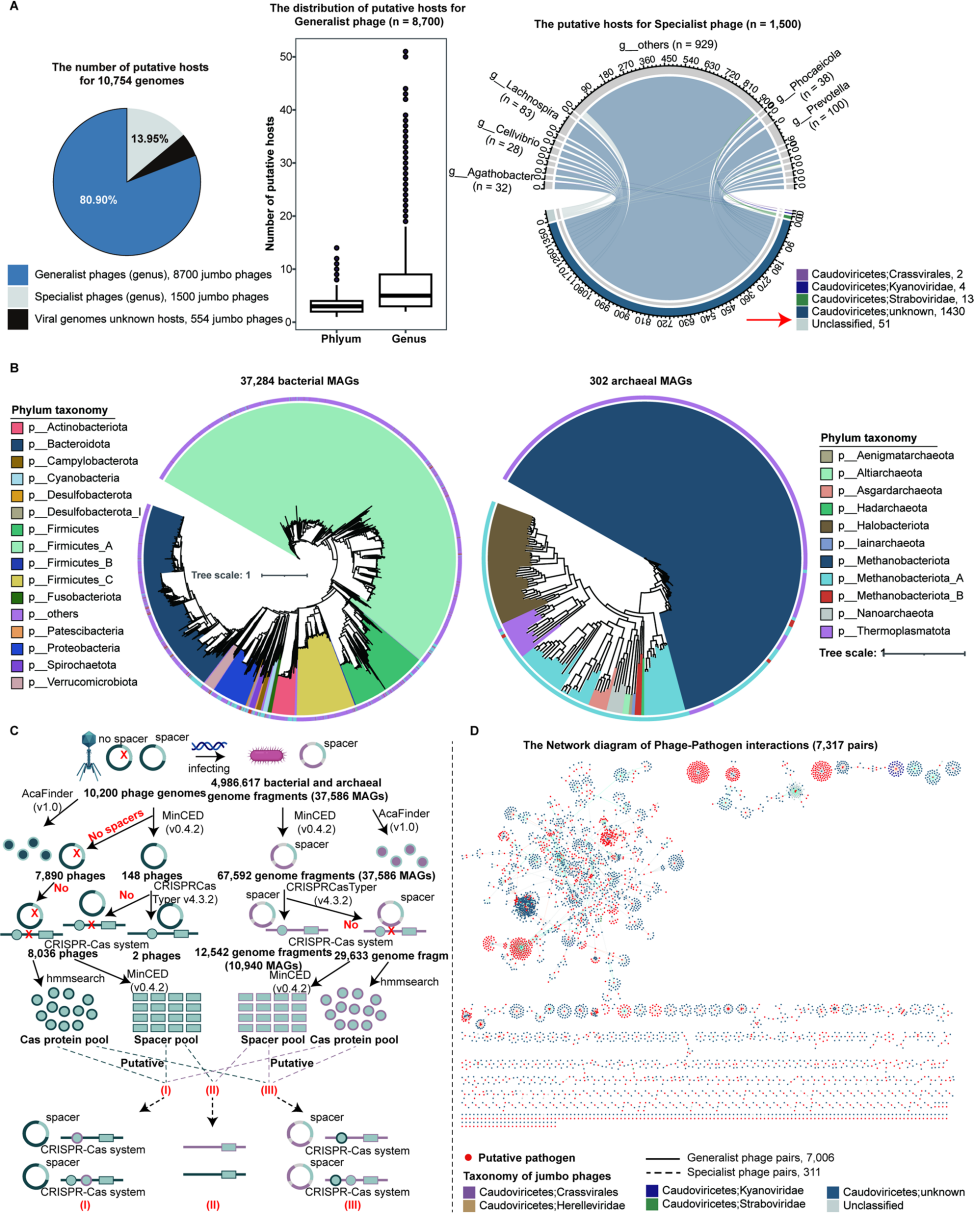
Identification of potential bacterial and archaeal hosts of jumbo phages using CRISPR spacer analysis. (**A**) The proportions of generalist and specialist jumbo phages in 10,754 jumbo phage genomes (pie chart), the distribution of the numbers of putative hosts at the phylum and genus levels for 8,700 generalist jumbo phages (box plot), and the distribution of putative bacterial hosts for 1,500 specialist jumbo phages (cycle diagram). (**B**) A phylogenetic tree of putative hosts (bacterial MAGs and archaeal MAGs) of jumbo phages. The outer circles represent the phylum-level taxonomy of host genomes. (**C**) The hypothesis for the interactions of jumbo phages with their bacterial or archaeal hosts without complete CRISPR-Cas systems and CRISPR-associated proteins. (**D**) A network diagram of phage-pathogen interaction for jumbo phages. Red dots represent putative pathogens, and other colors represent different taxonomies of jumbo phage genomes. The lines represent the generalist and specialist types

Matching bacterial or archaeal CRISPR spacer sequences to phage genomes has been one of the most important approaches to determining the host-parasite relationships between phages and bacteria or archaea [31]. Here, 10,200 (94.85%) of 10,754 jumbo phage genomes matched at least one CRISPR spacer sequence from one or multiple known bacteria or archaea (Additional file 6: Table S5). Most of the jumbo phages (*n* = 8,700) targeted multiple bacterial or archaeal genera and phyla (generalist phages) (Fig. 4A). There were 1,500 (13.95%) jumbo phages only targeting one bacterial or archaeal genus (specialist phages), and the hosts of these specialist jumbo phages primarily belonged to *Prevotella* and *Lachnospira*, keystone bacteria in the gut microbiome.

We found that 10,200 jumbo phages were matched to 37,284 bacterial MAGs and 302 archaeal MAGs, suggesting the broad interactions between jumbo phages and bacteria or archaea (Fig. 4B). Several bacterial or archaeal host genomes had no complete CRISPR-Cas systems or CRISPR-associated proteins. However, all of these host genomes harbored CRISPR spacer sequences, suggesting the infection of host bacteria or archaea (Fig. 4C). Furthermore, we found many solo Cas proteins in host bacterial or archaeal genomes and jumbo phage genomes, and analogous repeat sequences also occurred in the predicted host and jumbo phage CRISPR spacers. Therefore, we hypothesized that bacterial or archaeal genomes might be able to form complete CRISPR-Cas systems by hijacking Cas proteins from phage genomes, or from the homologous recombination of host and phage genomes (Fig. 4C). However, this hypothesis needed to be further verified. Additionally, we found anti-CRISPR proteins (Acrs) in both host and phage genomes. This could protect the phages from cleavage through CRISPR-Cas systems from the host genomes.

Specific jumbo phages (*n* = 2,993) in the JPGD targeted pathogenic bacteria, including *Salmonella enterica*, *Porphyromonas gingivalis*, *Staphylococcus aureus*, and *Streptococcus pneumoniae* based on the PHI database (http://www.phi-base.org/, Fig. 4D). Interestingly, compared with specialist phages (4.91%), a high proportion (95.09%) of the generalist phages targeted pathogenic bacteria, which were classified as virulent phages (78.05%). Furthermore, some jumbo phages (*n* = 1,070) could target multiple pathogenic bacteria (Additional file 7: Table S6). These results suggest that jumbo phages in the JPGD might be an ideal tool for regulating gut microbial composition by lysing pathogenic bacteria.

### CRISPR spacer analysis reveals competitive networks among jumbo phages

**Fig. 5:**
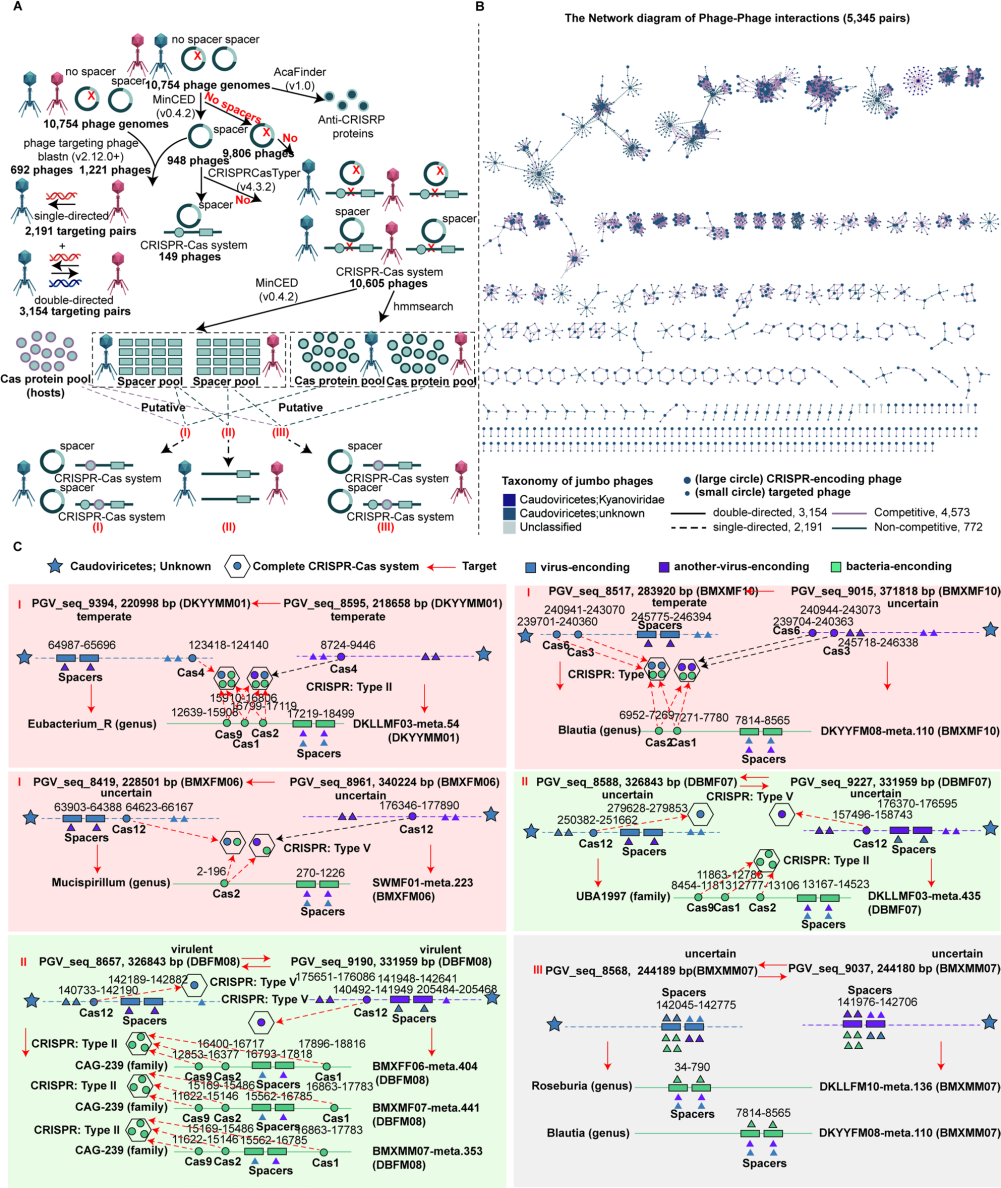
Interaction and competitive networks among jumbo phages shown by CRISPR spacer analysis. (**A**) The interaction patterns among jumbo phages inferred by analysis of the CRISPR-Cas systems. (**B**) The network diagram of phage-phage interactions. Dot sizes represent different phage types, and dot colors represent different phage taxonomies. The line colors represent competitive or non-competitive phages, and the line types represent single-directed and double-directed interaction phage pairs. (**C**) Three main interaction patterns of phages targeting phages detected in the same sample. Shadow diagrams show different interaction patterns of phages targeting phages. Diagrams with different colors indicate different jumbo phage and bacterial host genomes. Rectangles and circles represent spacers and Cas proteins, respectively. Triangles represent phage spacers that matched the genomic regions of targeted phages. The numbers indicate the regions of jumbo phage genomes, and the arrows indicate the targeting of one phage to another phage genome

Interestingly, some jumbo phages had both CRISPR spacers and intact functional CRISPR-Cas systems. These CRISPR-Cas systems could target host defenses and/or be involved in competing with other phages for the same bacterial hosts [3]. A total of 9,304 CRISPR spacers were detected in 948 jumbo phage genomes in the JPGD. Among these, 5,345 (57.45%) spacers were matched to 1,221 jumbo phage genomes, suggesting the existence of complex phage-phage interaction networks (Fig. 5A). A jumbo phage pair in which the spacers from one phage could match the other phage genome, but not vice versa, was defined as a single-directed interaction phage pair, and the jumbo phage pairs in which the spacers from two jumbo phages could match each other’s genomes were defined as double-directed interaction phage pairs. There was a higher proportion (59.00%, *n* = 3,154) of double-directed interaction phage pairs than single-directed interaction phage pairs, and 85.56% (*n* = 4,573) of phage-phage interaction pairs had the same bacterial or archaeal hosts and thus should be competitive interaction phages (Fig. 5B).

Additionally, most of the CRISPR-Cas systems identified in jumbo phage genomes lacked spacer acquisition units such as Cas1, Cas2, and Cas4 proteins. Furthermore, recognizable interfering genes, such as Cas3, Cas9, and Cas12 [3, 32] could not be detected in some jumbo phage genomes. However, the CRISPR-Cas systems in some jumbo phage genomes lacking spacers and recognizable interfering genes had CRISPR repeat sequences similar to those in their host genomes [3, 32]. To elucidate the underlying mechanism, we investigated the CRISPR-Cas systems in competing phages and their host bacteria (*n* = 19 pairs) in the same samples and proposed two possible patterns of the interactions among competing phages and their host bacteria. For the first pattern, the CRISPR-Cas systems of both competing phages and their host bacteria were incomplete and could not independently cleave foreign DNA fragments. The Cas proteins from competing phages were complementary to the CRISPR-Cas system of the bacterial hosts to form a complete and functional CRISPR-Cas system (Type I, Type II, and Type V systems) (Fig. 5C). From then on, the bacterial hosts could recognize the infection of both phages and deal with them. However, only the phage with spacer regions could recognize its competitor phage that had no spacer regions and cleaved it. For the second pattern, two competing phages and their bacterial host had their own intact CRISPR-Cas systems that could independently cleave foreign DNA fragments. Bacterial hosts could recognize the infection of both phages, and the two competing phages could recognize each other. We observed another phenomenon (III in Fig. 5C) where the genomes of two competitive phages and their bacterial hosts did not have a CRISPR-Cas system, but the spacer regions containing both viral and host bacterial sequences were present. We hypothesized that these foreign DNA fragments might be captured by homologous recombination. However, this hypothesis needed to be further verified. Notably, anti-CRISPR proteins (Acrs) that should help jumbo phages to segregate from their host bacterial genomes were identified in some jumbo phage genomes (Additional file 8: Table S7).

### Functions of jumbo phage genomes

**Fig. 6:**
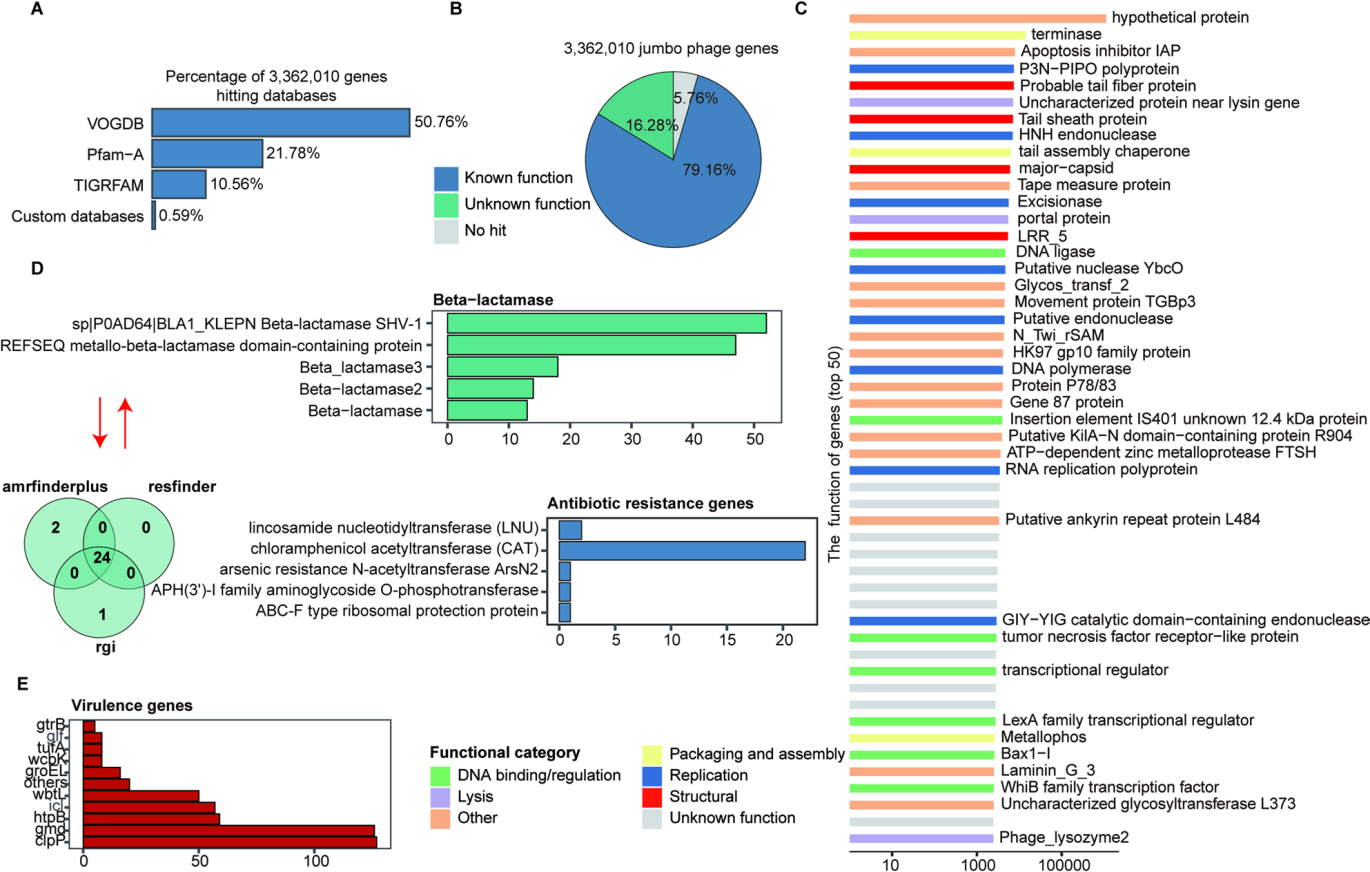
Functional annotation of jumbo phage genomes in the JPGD. **(A**) The percentage of jumbo phage genes hitting each of the four databases. (**B**) An overview of functional annotation of 3,362,010 jumbo phage genes. (**C**) Functional items with the numbers of jumbo phage genes in the top 50. The colors represent different functional categories. (**D**) Antibiotic resistance genes (ARGs) identified in jumbo phage genomes. ARGs were identified using Resfinder, AMRfinder, and RGI. The main categories of ARGs are shown, and the specific ARGs of putative beta-lactamases are indicated. (**E**) Virulence factor genes (VFGs) identified in jumbo phage genomes in the JPGD

Although potential functions of gut phages have been extensively reported [28, 66], the functional capacities of jumbo phages from different habitats are largely unknown. We identified 3,362,010 coding genes from 10,754 jumbo phage genomes and annotated these genes with TIGRFAM, Pfam-A, VOGDB, and a custom viral structural protein database (Fig. 6A). Overall, 5.76% of jumbo phage genes had no significant hits in the above databases; 16.28% were matched to hits in the databases but could not be assigned to any biological function categories, and 79.16% of phage genes could be functionally annotated (Fig. 6B). We further classified these genes into functional items. Most of the annotated genes belonged to hypothetical proteins. Functional items related to protein structure, assembly and packaging, DNA replication and transcription, and lysis were enriched by annotated genes (Fig. 6C). These functional items were typical functional capacities of phages.

We further searched for homologs of ARGs in jumbo phage genomes in the JPGD using Resfams, AMRFinder, and RGI. A total of 27 non-redundant ARGs were identified. Among these, 24 ARGs were identified by all three programs; two ARGs were specifically identified by AMRFinder, and one ARG was specifically detected by RGI. Most of these 27 ARGs belonged to chloramphenicol acetyltransferase (Fig. 6D). Notably, we identified 144 variants of the *β-lactamase* gene in jumbo phage genomes; these are related to the resistance of bacterial hosts to *β-lactam* antibiotics such as penicillin, cephalosporins, and cephalosporanes. The results suggested that jumbo phages could be important carriers or transmitters of ARGs.

Phages can also spread VFGs between bacterial strains, including genes encoding toxins that may cause diseases such as diphtheria, cholera, dysentery, botulism, staphylococcal exfoliative skin syndrome, noma, and scarlet fever [24, 67–69]. We identified 484 virulence factor genes (VFGs) from the JPGD using the VFDB database, including some VFGs containing high numbers of variants such as *clpP*, *gmd*, *htpB*, *icl*, and *wbtL* (Fig. 6E).

### AMGs in jumbo phage genomes

**Fig. 7:**
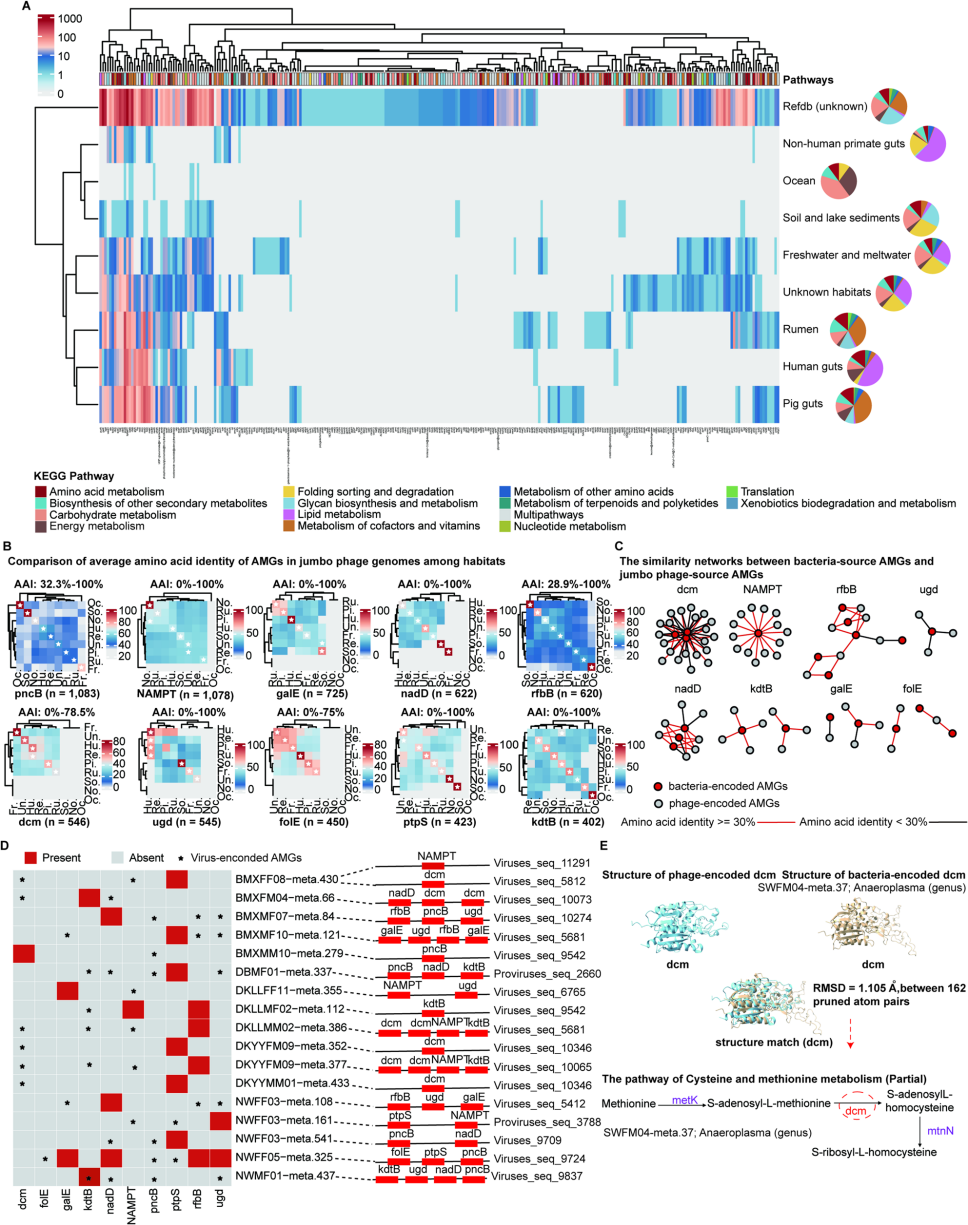
Auxiliary metabolic genes (AMGs) identified in jumbo phage genomes and their predicted functional roles in the metabolism of the bacterial hosts. (**A**) The distribution of AMGs obtained from jumbo phage genomes in the JPGD among different habitats. KEGG pathways enriched by AMGs are colored according to the legends. (**B**) Comparison of average amino acid identity (mAAI) of AMGs in jumbo phage genomes among different habitats. The stars represent the highest value of mAAI in a column. (**C**) Similarity networks between jumbo phage-sourced AMGs and bacterial-sourced AMGs. The dots with different colors represent AMGs from two different sources, and lines with different colors represent amino acid identity. (**D**) Complementarity of jumbo phage AMGs with bacterial host genomes (MAGs). Dashed lines indicate the inferred bacterial hosts of the corresponding jumbo phages. The presence and absence of the homologs of jumbo phage AMGs in MAGs (left) are indicated by red and gray, respectively. The asterisks indicate the AMGs from corresponding jumbo phages. For jumbo phage genomes (right), the genomic contexts of AMGs (red) are shown. (**E**) The *dcm* gene as an example of an AMG involved in the cysteine and methionine metabolism of bacterial hosts. The similarity of the 3D structures of phage-sourced *dcm* and bacterial-sourced *dcm* was analyzed, and a partial pathway of cysteine and methionine metabolism is shown

Viruses can directly influence the metabolism of their bacterial hosts through their AMGs [8]. AMGs in jumbo phage genomes were identified using VIBRANT (v1.2.1) and DRAMv (v1.4.4). A total of 14,854 potential AMGs were detected in 5,068 jumbo phage genomes, of which 3,402 contained more than two AMGs. These 14,854 AMGs were functionally annotated using the KEGG database and were largely classified into the pathways of amino acid metabolism, cofactor and vitamin metabolism, carbohydrate metabolism, glycan biosynthesis and metabolism, and lipid metabolism (Fig. 7A). The categories of AMGs carried by jumbo phage genomes from different habitat types were distinct. The pathways of carbohydrate metabolism, amino acid metabolism, energy metabolism, and biosynthesis of other secondary metabolites were enriched by AMGs detected in jumbo phage genomes from all habitat types. However, the pathway of folding sorting and degradation was specifically enriched by AMGs in jumbo phage genomes from soil, freshwater, and marine habitats. Significantly higher numbers of AMGs in jumbo phage genomes from the human gut and non-human primate gut were enriched in lipid metabolism than those in jumbo phage genomes from other habitats. AMGs in jumbo phage genomes from the rumen and pig gut were significantly enriched in the pathway of metabolism of cofactors and vitamins (Additional file 9: Table S8).

We also found some AMGs that were associated with methane, carbon, nitrogen, and sulfur cycling in jumbo phage genomes. For example, jumbo phages from freshwater samples carried the AMGs *pmoC-amoC* that are involved in methane production. The *pmoC* gene from jumbo phage genomes could enhance methane oxidation in bacteria, thereby affecting the emission of methane into the atmosphere [25]. DNA methyltransferases (*dcm*), cysteine synthases (*CysK*), and glutamine synthetase (*glnA*) were also identified in jumbo phage genomes. The *dcm* gene is involved in methionine degradation, and *CysK* is involved in the assimilatory sulfate reduction; both have been reported to be associated with the sulfur cycle [15]. The *glnA* gene catalyzes the production of glutamine from glutamate and is associated with the nitrogen cycle [15]. These findings suggest that jumbo phages may drive biogeochemical cycling through their AMGs.

Previous studies have shown that habitat type has an important effect on the variation of viral AMGs [15, 70]. We selected AMGs from those ranked in the top 10 for a comparative analysis of protein AAI among different habitats. The protein sequences of AMGs from different habitats showed certain similarities, suggesting comparable functions among habitats. In particular, protein sequences of AMG variants from the same habitats were more similar than those from different habitat types (Fig. 7B). We further compared the sequence similarity of jumbo phage AMG proteins with homologs from bacteria. The results indicated that the protein sequences of jumbo phage AMGs showed certain similarities with corresponding homologs from bacteria (Fig. 7C), confirming the hypothesis of similar functions in metabolism between jumbo phage AMGs and homologous bacterial genes.

To further investigate the auxiliary metabolic functions of jumbo phage AMGs, we analyzed the distribution of jumbo phage AMGs in the genomes of their corresponding bacterial hosts by focusing on AMGs ranked in the top 10 (Fig. 7D). Most of these AMGs had no identified homologs in the genomes (MAGs) of their corresponding bacterial hosts. This was consistent with the auxiliary metabolic functions of jumbo phage AMGs relative to the bacterial hosts. As an example, *dcm*, an important gene that encodes a key enzyme in the methylation of S-adenosyl-L-methionine to S-adenosyIL-homocysteine in cysteine and methionine metabolism, was identified in many jumbo phage genomes but not in the genomes of their bacterial hosts. Furthermore, the predicted 3D structures of jumbo phage AMG proteins such as *dcm* using ColabFold were highly similar to the 3D structures of their homologs from bacteria, for example, MAG: SWFM04-meta.37 with an RMSD of 1.105 μm, suggesting its auxiliary metabolic function. Notably, we identified the other two key genes (*metK* and *mtnN*) involved in the catalysis of methionine to S-adenosyl-L-methionine (*metK*) and S-adenosyIL-homocysteine to S-ribosyl-L-homocysteine in the pathway of cysteine and methionine metabolism in the corresponding bacterial genome (MAG: SWFM04-meta.37) (Fig. 7E). This result suggests that AMGs carried by jumbo phages may compensate for the metabolic capacity of the bacterial hosts. We identified homologous genes of several AMGs in the genomes of their bacterial hosts. The effect of these AMGs on host metabolism is currently unknown. We suspect that they may enhance the metabolic flux of host bacteria by overexpressing these genes.

### JPGD has significantly expanded the diversity of Caudoviricetes

To remove the effect of fragmented phage genomes on the analysis of evolutionary relationships among jumbo phage genomes, we selected 3,341 jumbo phage genomes with 100% genome integrity (intact jumbo phage genomes) using CheckV (v0.8.1) (Additional file 1: Figure S3A). These intact jumbo phage genomes were detected in all habitat types, and the highest number of intact jumbo phage genomes occurred in the pig gut (*n* = 645). We then predicted tRNAs from 3,341 intact jumbo phage genomes, and tRNAs were detected in 2,494 of these genomes. The number of detected tRNAs per genome increased with genome size (Spearman *r* = 0.34, *P* < 0.001) (Additional file 1: Figure S3B). Up to 70 tRNAs with different sequences from the corresponding host bacterial tRNAs were found in a jumbo phage genome.

**Fig. 8:**
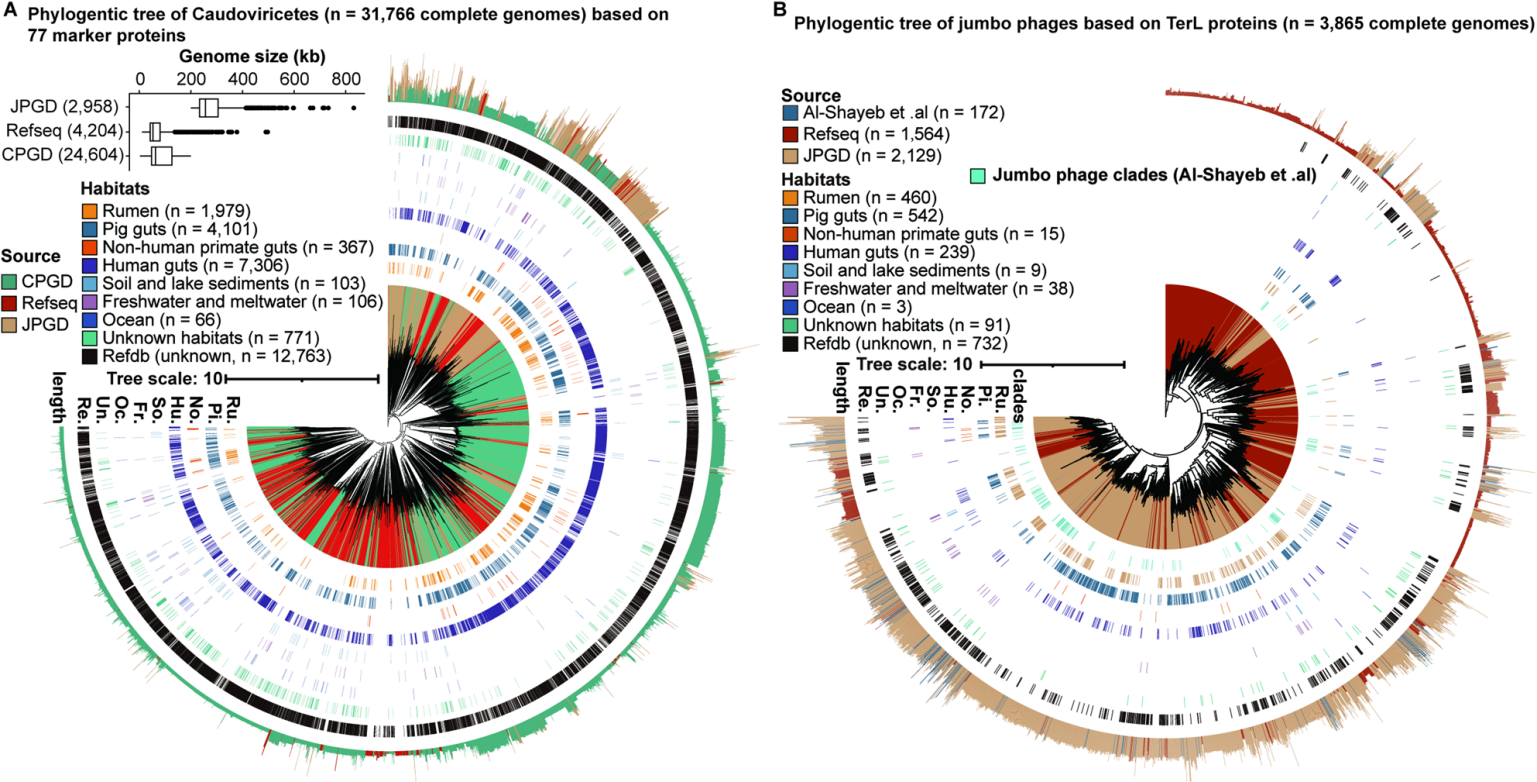
Phylogeny analysis of Caudoviricetes and jumbo phages based on complete genomes. (**A**) Phylogenetic tree of Caudoviricetes genomes based on 77 marker proteins. The phylogenetic tree was plotted using iToL. Branch colors indicate the sources of lineages. Refseq: red; JPGD: yellow; and CPGD: green. The outer bars represent the genome sizes of phages, and the outer bar colors indicate the different sources of phages. The colored inner cycles show different habitats. In the box plot, the center lines, upper and lower bounds, and upper and lower whiskers show median values, 25th and 75th quantiles, and the largest and smallest non-outlier values, respectively. (**B**) Phylogenetic analysis of jumbo phages with complete genomes based on *TerL* proteins. The outer circle and branch colors represent jumbo phage sources, and the colored inner circles indicate the habitats of the jumbo phages

Caudoviricetes is a class of tailed dsDNA phages distributed among various environments, and it contains a high proportion of jumbo phages. To explore the evolutionary relationships of jumbo phage genomes belonging to Caudoviricetes in different habitat types, we constructed a phylogenetic tree based on tandem comparisons of 77 protein-coding marker genes. To avoid the effect of fragmented genomes, we used complete jumbo phage genomes of Caudoviricetes from the JPGD (*n* = 3,342) and Refseq (*n* = 4,463) and complete phage genomes of Caudoviricetes with genome sizes ≤ 200 kb from the CPGD (*n* = 4,779,213). After removing those genomes with less than three marker genes or < 5% of aligned sequence in the multiFASTA alignment, a total of 31,766 phage genomes of Caudoviricetes (JPGD: 2,958; Refseq: 4,204; and CPGD: 24,604) were used to construct the phylogenetic tree. Based on the cumulative branch lengths, phage genomes of Caudoviricetes in the JPGD and CPGD covered 14.03% and 81.03% of the total phylogenetic diversity (PD), respectively, and represented all major lineages in the tree. Moreover, the jumbo phage genomes were clustered at the top-right corner of phylogenetic tree (Fig. 8A). Caudoviricetes genomes from the pig gut, human gut, rumen, and non-human primate gut covered 19.40%, 17.89%, 15.42%, and 1.33% of the total PD, respectively, and were evenly distributed among viral taxa. Notably, after removing the genomes from Refseq and IGN, the remaining complete genomes from the JPGD and CPGD comprised 51.19% of the total PD, indicating that viral genomes in the JPGD and PGD have significantly expanded the PD of Caudoviricetes and covered most Caudoviricetes lineages. Interestingly, members of Caudoviricetes, including both jumbo phages and conventional phages, appeared to be broadly distributed among various habitat types.

To validate jumbo phages were formed as the result of recent genome expansion of phages through stable ongoing evolution [3], we constructed a phylogenetic tree based on *TerL* proteins (*n* = 3,865) of 2,988 complete jumbo phage genomes, comprising 1,426 genomes from Refseq, 1,414 genomes from the JPGD, and 148 genomes from the study of Al-Shayeb et al. [3]. The topology of the phylogenetic tree varied slightly across datasets. As described earlier, jumbo phage genomes tended to cluster (Fig. 8B), suggesting that the large genome size of jumbo phages is a relatively stable feature. Each clade contained jumbo phage genomes from a wide variety of environments, demonstrating the conservation of jumbo phage genomes and the diversity of their hosts. Based on cumulative branch lengths, the *TerL* proteins of jumbo phage genomes from the JPGD and the study of Al-Shayeb et al.. covered 58.34% and 8.90%, respectively, of the PD, and these jumbo phage genomes were distributed in all major lineages of the phylogenetic tree. Furthermore, the *TerL* proteins of jumbo phage genomes from the pig gut, rumen, human gut, and non-human primate gut covered 17.90%, 15.54%, 10.71%, and 1.11% of the total PD, respectively, and were evenly distributed among viral taxa (Fig. 8B). Similarly, after removing the genomes from Refseq and IGN, the *TerL* proteins of jumbo phage genomes from the JPGD covered 37.88% of the total PD.

## Discussion

Jumbo phages are widespread in the Earth’s ecosystems, and they have been detected in various habitat types, ranging from animal guts to soil and freshwater ecosystems [3, 25]. However, previous studies [3, 24] have been unable to comprehensively characterize the diversity, genomic structure, and distribution of jumbo phages in microecosystems due to the relative scarcity of jumbo phage genomes. We constructed a database containing 10,754 jumbo phage genomes by using public metagenomic sequencing datasets, the PGV genome database, and public viral genome databases covering various habitat types. We characterized the composition and diversity, genomic structure, potential function capacities, bacterial hosts, and the distribution of jumbo phages in different habitats. This study provides important resources for further investigating the roles of jumbo phages in microecosystems.

We found that the proportions of jumbo phages in phages or the microbial community were particularly low (0.18%), and some datasets did not identify jumbo phage genomes. We hypothesized that the fragmentation of jumbo phage genomes in metagenomic sequencing analysis may affect the detection of jumbo phages. Additionally, structural proteins of jumbo phages might be difficult to be discovered due to their low homology with known phage proteins, especially under the condition of the utilization of alternative genetic codes. Compared with current viral genome databases, the JPGD constructed in this study provided the largest number of jumbo phage genomes and significantly expanded the known diversity of jumbo phages.

We found that some jumbo phage genomes showed potential coevolution with their habitats. This was consistent with the report that habitat types can influence the distribution and diversity of jumbo phages [3] and further emphasized the importance of jumbo phages in maintaining the stability of the microecosystem. However, further analyses should be performed to confirm this coevolution relationships of jumbo phage genomes with habitats using more dataset from large sample size. We systematically discussed the genome features of jumbo phages, such as alternative codon usage, tRNAs, AMGs, and ARGs. Although these features are not unique to jumbo phages, compared to other phages, whether the expanded genome size of jumbo phages should lead to some differences in the abundance, diversity, and potential complexity of these genome features have been largely unknown. Therefore, it is worth discussing these genomic features in jumbo phages. A notable feature of jumbo phage genomes was their low coding density and the fragmentation of many predicted proteins due to the utilization of alternative genetic codes [24]. Jumbo phage genomes using alternative genetic codes displayed a lower protein coding density than phages using the standard genetic code (11). The adoption of alternative genetic codes by jumbo phages could not only maintain the compactness of their genomic structure but also ensure efficient protein coding. Previous studies have reported that genes with stop codon recoding also had higher numbers of SNPs per nucleotide than genes without stop codon recoding, and the gene signatures of alternative genetic codes can increase the stability of jumbo phages in organisms [22, 24]. Consistent with the results of previous studies [3, 24], jumbo phage genomes in the JPGD encoded many tRNAs and suppressor tRNAs, which might improve the translation of proteins and ensure the recoding of stop codons. Phages recoding the stop codon TAG usually encoded a suppressor tRNA with a CTA anticodon. Not all jumbo phages recoding stop codons also had a suppressor tRNA. We inferred that these jumbo phages might use suppressor tRNAs from their bacterial hosts or competing phages in the recoding of stop codons. However, further experiments are needed to validate this hypothesis.

In the JPGD, a higher proportion of jumbo phages than other phages tended to target multiple bacterial genera, and their bacterial hosts were spread across multiple phyla. Furthermore, most of the jumbo phages in the JPGD were virulent phages, and some jumbo phages could target some common pathogens such as *Salmonella enterica* and *Porphyromonas gingivalis*. This suggested that jumbo phages in the JPGD were an important resource for developing phage therapy targeting pathogens. As reported in a previous study [3], some jumbo phage genomes identified in this study contained intact and functional CRISPR-Cas systems. These CRISPR-Cas systems may respond to host defenses as well as to competition among phages for the same bacterial hosts. Through investigation of the CRISPR-Cas system of jumbo phage genomes and their bacterial hosts, we suggest that the components of the CRISPR-Cas systems in the genomes of jumbo phages and their bacterial hosts could complement each other to form intact and functional CRISPR-Cas systems. A previous study reported that phages could use the components of the CRISPR-Cas system in bacterial hosts to edit foreign DNA [32]. These results indicate that jumbo phage genomes in the JPGD could act as a library for developing genome editing tools. Moreover, these jumbo phage genomes encoded anti-CRISPR proteins (Acrs) that could facilitate the segregation of their replicated genome sequences from the host genomes.

We identified hundreds of hypothetical proteins in jumbo phage genomes. Previous studies have suggested that hypothetical proteins were precursor proteins for functionally important proteins of phages and could ensure the successful replication of phages when faced with host defenses [24]. These hypothetical proteins may also be important for expanding the range of bacterial hosts [24]. Viruses have been recognized as crucial carriers and transmitters of ARGs [19, 71]. However, the diversity and abundance of ARGs are generally lower in the virome than in bacteria. We identified a small number of ARGs in jumbo phage genomes. Previous studies have indicated the importance of viral AMGs in the metabolism of bacterial hosts [14, 15]. However, the distribution and functional capacities of AMGs in jumbo phage genomes are largely unknown. We observed that AMGs in jumbo phage genomes differed among habitat types and were associated with methane, carbon, nitrogen, and sulfur cycling.

Caudoviricetes is a class of tailed dsDNA phages found in various environments and is currently the largest class of phages. Jumbo phages belonging to Caudoviricetes have rarely been reported. This study identified novel jumbo phage genomes belonging to the Caudoviricetes class. This has significantly increased the known diversity of Caudoviricetes. Consistent with the relatively stable feature of the large genome size [3], the jumbo phage genomes were clustered in the phylogenetic trees. Furthermore, each clade contained jumbo phage genomes from a wide variety of environmental types, demonstrating the conservation of jumbo phage genomes and the diversity of their hosts.

## Conclusions

This study constructed a comprehensive jumbo phage genome database containing 10,754 genomes, which greatly expanded the diversity of jumbo phages, especially Caudoviricetes. Based on this database, we highlighted the genomic structure of jumbo phage genomes, especially elucidated the features of recoding of stop codons and the CRISPR-Cas systems in jumbo phage genomes. We also revealed potential bacterial hosts for jumbo phages and the competitive networks among phages. This study also emphasized the potential roles of jumbo phages in the metabolism of bacterial host through AMGs, suggested the wide existence of jumbo phages in various microecosystems, and gave new insights into the diversity and genomic structure of gut jumbo phages. This study provided an important source for the future studies on the interaction between jumbo phages and bacteria.

## Supplementary Information

Below is the link to the electronic supplementary material.

Supplementary Material 1: Additional file 1: Figure S1. Identification and distribution of jumbo phage genomes. A The geographical distribution of 571 pig gut microbial samples. The countries where pig gut metagenomic sequencing data were from are highlighted with green color. The stacked bars with different colors and numbers represent the exact number of pig gut samples. B The percentage and assembly assessment of jumbo phages. The pies represent the percentages of jumbo phages in all phage genomes and jumbo phages from the PGV dataset in the PJPGD. The uniformed sequencing depth (right) indicated the accuracy of jumbo phage genome assembly

Supplementary Material 2: Additional file 1: Figure S2. Lifestyle, and encoding suppressor tRNA of 385 jumbo phage genomes using alternative genetic codes. A The lifestyle of 385 jumbo phages using alternative genetic codes. The pie shows the proportion of temperate and virulent phages for 385 jumbo phage genomes. B The proportion of jumbo phage genomes with encoding tRNAs, suppressor tRNAs, and intron in 385 jumbo phage genomes. C Schematics represent the introns and tRNA in jumbo phage genomes

Supplementary Material 3: Additional file 1: Figure S3. The tRNA distribution of complete jumbo phages, and phylogenetic tree of crAssphages and Lakphages. A The proportion of complete genomes in the JPGD in different habitats. B The number of coding tRNA from 2,493 complete jumbo phage genomes in different habitats. Different dots represent different jumbo phage genomes, and different colors indicate jumbo phages from different habitats

Supplementary Material 4: Additional file 2: Table S1. The data sources used in this study for jumbo phage genomes

Supplementary Material 5: Additional file 3: Table S2. The summarized numbers of viruses, phages, and jumbo phages identified in each study

Supplementary Material 6: Additional file 4: Table S3. The detailed information on genome length, completeness, lifestyle, taxonomy, and genetic codes for 10,754 jumbo phage genomes in the JPGD

Supplementary Material 7: Additional file 5: Table S4. The exact P values for co-evaluation of jumbo phage genomes with habitat types in the JPGD

Supplementary Material 8: Additional file 6: Table S5. The bacterial hosts of 10,754 jumbo phages in the JPGD

Supplementary Material 9: Additional file 7: Table S6. The interactions of generalist and specialist jumbo phages in the JPGD with pathogens

Supplementary Material 10: Additional file 8: Table S7. The distribution and competing types of phage-phage interactions in the JPGD

Supplementary Material 11: Additional file 9: Table S8. The detailed information on auxiliary metabolic genes (AMGs) in jumbo phage genomes

## Data Availability

Jumbo phage genomes in the JPGD can be accessed and downloaded without any restriction at https://doi.org/10.5281/zenodo.14974448. The codes for jumbo phage analysis are also available at https://doi.org/10.5281/zenodo.14974448. All other datasets are available in the supplementary materials.

## Abbreviations

JPGD: Jumbo phage genome database
IGN: An integrated public viral database
NT: Nucleotide Sequence Database
MAGs: Metagenomic assembled genomes
AMGs: Auxiliary metabolic genes
ARGs: Antimicrobial resistance genes
VFGs: Virulence factor genes
ANI: Average nucleotide identity
AAI: Average amino acid identity
PD: Phylogenetic diversity
ORF: Open reading frame
dsDNA: double-stranded DNA
Acrs: anti-CRISPR proteins
