# Supplementary figures and images for: Large-scale genomic analysis of jumbo phages: coevolution, genome architecture, and host interaction mechanisms
Source: Anim Microbiome. 2026 Feb 24;8:32. doi: 10.1186/s42523-026-00534-z (PMC13037316; doi:10.1186/s42523-026-00534-z)

A

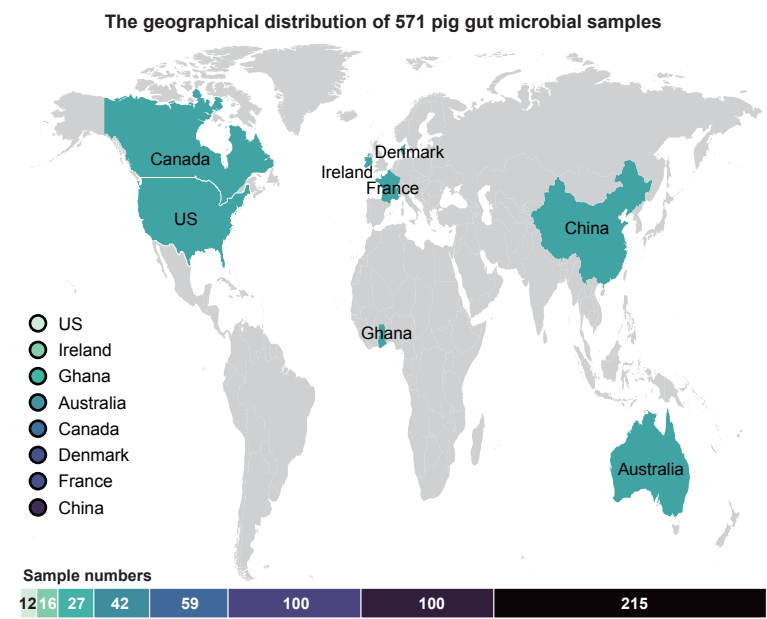

B

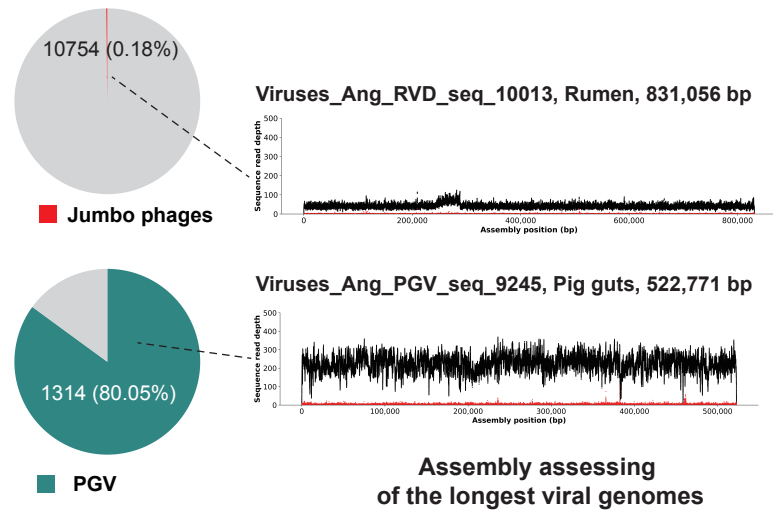

Supplement: Supplementary file 1 — Supplementary Material 1: Additional file 1: Figure S1. Identification and distribution of jumbo phage genomes. A The geographical distribution of 571 pig gut microbial samples. The countries where pig gut metagenomic sequencing data were from are highlighted with green color. The stacked bars with different colors and numbers represent the exact number of pig gut samples. B The percentage and assembly assessment of jumbo phages. The pies represent the percentages of jumbo phages in all phage genomes and jumbo phages from the PGV dataset in the PJPGD. The uniformed sequencing depth (right) indicated the accuracy of jumbo phage genome assembly [file 42523_2026_534_MOESM1_ESM.pdf]
