# Supplementary material for: Large-scale genomic analysis of jumbo phages: coevolution, genome architecture, and host interaction mechanisms
Source: Anim Microbiome. 2026 Feb 24;8:32. doi: 10.1186/s42523-026-00534-z (PMC13037316; doi:10.1186/s42523-026-00534-z)
Supplement: Supplementary file 2 — Supplementary Material 2: Additional file 1: Figure S2. Lifestyle, and encoding suppressor tRNA of 385 jumbo phage genomes using alternative genetic codes. A The lifestyle of 385 jumbo phages using alternative genetic codes. The pie shows the proportion of temperate and virulent phages for 385 jumbo phage genomes. B The proportion of jumbo phage genomes with encoding tRNAs, suppressor tRNAs, and intron in 385 jumbo phage genomes. C Schematics represent the introns and tRNA in jumbo phage genomes [file 42523_2026_534_MOESM2_ESM.pdf]

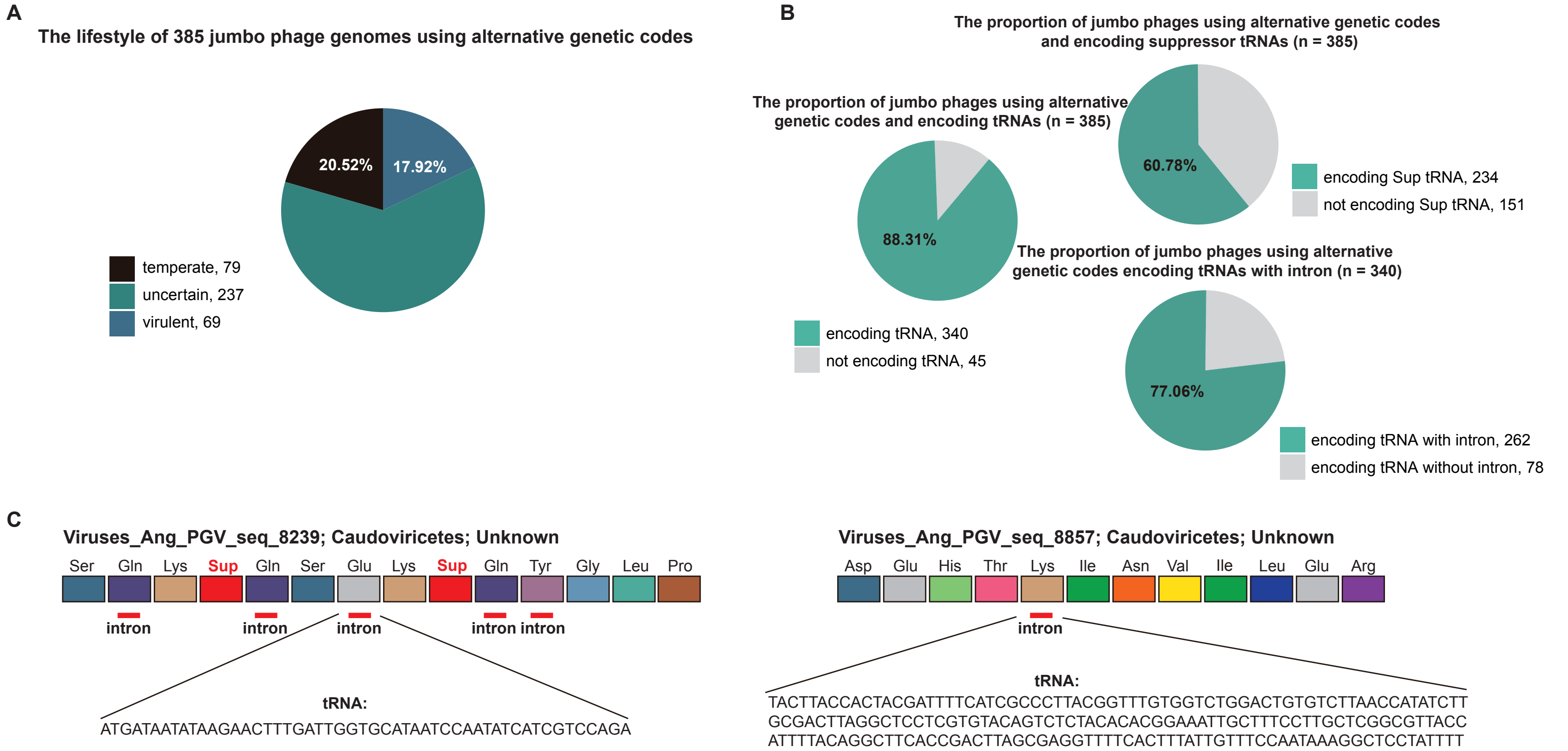
A
The lifestyle of 385 jumbo phage genomes using alternative genetic codes
20.52%
17.92%
temperate, 79
uncertain, 237
virulent, 69
B
The proportion of jumbo phages using alternative genetic codes and encoding suppressor tRNAs (n = 385)
The proportion of jumbo phages using alternative genetic codes and encoding tRNAs (n = 385)
60.78%
encoding Sup tRNA, 234
not encoding Sup tRNA, 151
88.31%
The proportion of jumbo phages using alternative genetic codes encoding tRNAs with intron (n = 340)
encoding tRNA, 340
not encoding tRNA, 45
77.06%
encoding tRNA with intron, 262
encoding tRNA without intron, 78
C
Viruses_Ang_PGV_seq_8239; Caudoviricetes; Unknown
Ser Gln Lys Sup Gln Ser Glu Lys Sup Gln Tyr Gly Leu Pro
intron intron intron intron intron
tRNA:
ATGATAATATAAGAACTTTGATTGGTGCATAATCCAATATCATCGTCCAGA
Viruses_Ang_PGV_seq_8857; Caudoviricetes; Unknown
Asp Glu His Thr Lys Ile Asn Val Ile Leu Glu Arg
intron
tRNA:
TACTTACCACTACGATTTTCATCGCCCTTACGGTTTGTGGTCTGGACTGTGTCTTAACCATATCTT
GCGACTTAGGCTCCTCGTGTACAGTCTCTACACACGGAAATTGCTTTCCTTGCTCGGCGTTACC
ATTTTACAGGCTTCACCGACTTAGCGAGGTTTTCACTTTATTGTTTCCAATAAAGGCTCCTATTTT
