# Supplementary material for: Large-scale genomic analysis of jumbo phages: coevolution, genome architecture, and host interaction mechanisms
Source: Anim Microbiome. 2026 Feb 24;8:32. doi: 10.1186/s42523-026-00534-z (PMC13037316; doi:10.1186/s42523-026-00534-z)
Supplement: Supplementary file 3 — Supplementary Material 3: Additional file 1: Figure S3. The tRNA distribution of complete jumbo phages, and phylogenetic tree of crAssphages and Lakphages. A The proportion of complete genomes in the JPGD in different habitats. B The number of coding tRNA from 2,493 complete jumbo phage genomes in different habitats. Different dots represent different jumbo phage genomes, and different colors indicate jumbo phages from different habitats [file 42523_2026_534_MOESM3_ESM.pdf]

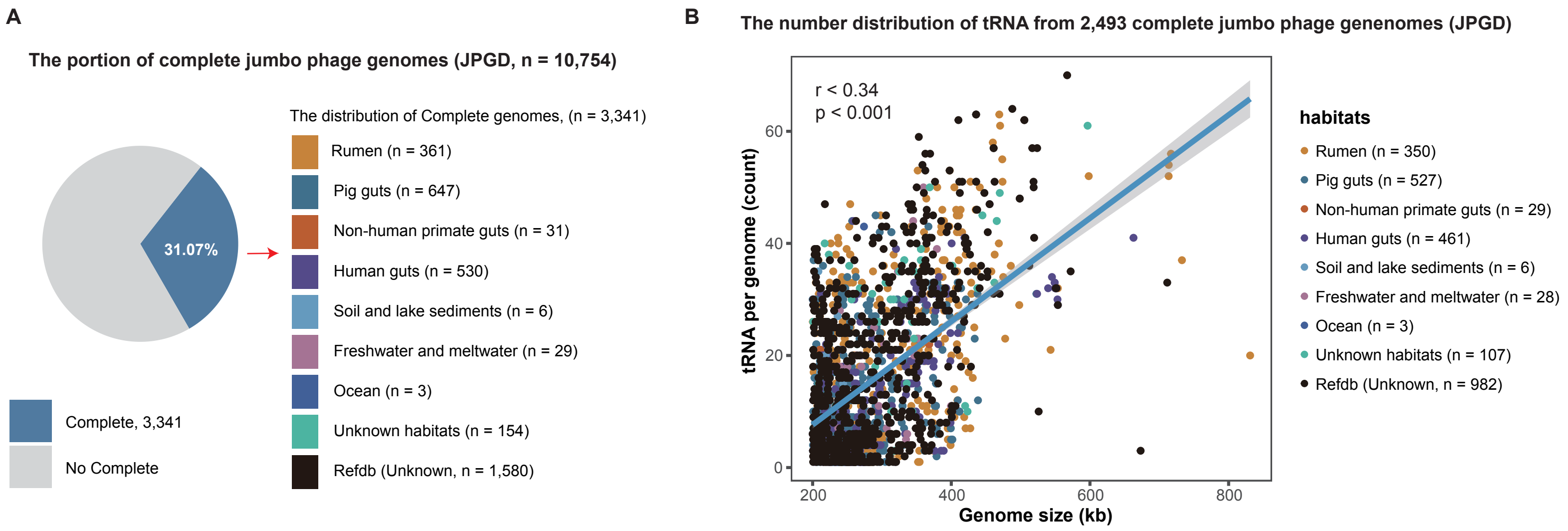
A
The portion of complete jumbo phage genomes (JPGD, n = 10,754)
31.07%
The distribution of Complete genomes, (n = 3,341)
Rumen (n = 361)
Pig guts (n = 647)
Non-human primate guts (n = 31)
Human guts (n = 530)
Soil and lake sediments (n = 6)
Freshwater and meltwater (n = 29)
Ocean (n = 3)
Unknown habitats (n = 154)
Refdb (Unknown, n = 1,580)
Complete, 3,341
No Complete
B
The number distribution of tRNA from 2,493 complete jumbo phage genenomes (JPGD)
r < 0.34
p < 0.001
tRNA per genome (count)
Genome size (kb)
habitats
Rumen (n = 350)
Pig guts (n = 527)
Non-human primate guts (n = 29)
Human guts (n = 461)
Soil and lake sediments (n = 6)
Freshwater and meltwater (n = 28)
Ocean (n = 3)
Unknown habitats (n = 107)
Refdb (Unknown, n = 982)
